# Oxr1 Is Essential for Protection against Oxidative Stress-Induced Neurodegeneration

**DOI:** 10.1371/journal.pgen.1002338

**Published:** 2011-10-20

**Authors:** Peter L. Oliver, Mattéa J. Finelli, Benjamin Edwards, Emmanuelle Bitoun, Darcy L. Butts, Esther B. E. Becker, Michael T. Cheeseman, Ben Davies, Kay E. Davies

**Affiliations:** 1 Medical Research Council Functional Genomics Unit, Department of Physiology, Anatomy, and Genetics, University of Oxford, Oxford, United Kingdom; 2 Medical Research Council Harwell, Harwell, United Kingdom; 3 Wellcome Trust Centre for Human Genetics, Oxford, United Kingdom; University of Minnesota, United States of America

## Abstract

Oxidative stress is a common etiological feature of neurological disorders, although the pathways that govern defence against reactive oxygen species (ROS) in neurodegeneration remain unclear. We have identified the role of oxidation resistance 1 (Oxr1) as a vital protein that controls the sensitivity of neuronal cells to oxidative stress; mice lacking Oxr1 display cerebellar neurodegeneration, and neurons are less susceptible to exogenous stress when the gene is over-expressed. A conserved short isoform of Oxr1 is also sufficient to confer this neuroprotective property both *in vitro* and *in vivo*. In addition, biochemical assays indicate that Oxr1 itself is susceptible to cysteine-mediated oxidation. Finally we show up-regulation of Oxr1 in both human and pre-symptomatic mouse models of amyotrophic lateral sclerosis, indicating that Oxr1 is potentially a novel neuroprotective factor in neurodegenerative disease.

## Author Summary

Oxygen is vital for life, but it can also cause damage to cells. Consequently, protective proteins (antioxidants) are utilised to maintain the fine balance between oxygen metabolism and the production of potentially toxic reactive oxygen species (ROS). If this balance is not maintained, oxidative stress occurs and excess ROS are generated, causing damage to DNA, proteins, and lipids. The brain is particularly susceptible to oxidative stress, and ROS–induced damage is a common feature of all major neurodegenerative disorders, including amyotrophic lateral sclerosis (ALS) and Parkinson's disease (PD). However, the molecular mechanisms of ROS defence in neurons are still under investigation. Here we describe the characterisation of oxidation resistance 1 (*Oxr1*), a gene previously shown to be induced under oxidative stress. We show both in mice and in cells that loss of Oxr1 causes cell death and that increasing protein levels can protect against ROS. In addition, Oxr1 is over-expressed in the spinal cord in ALS patients, as well as in a pre-symptomatic ALS mouse model. These data demonstrate for the first time that Oxr1 is vital for the protection of neuronal cells against oxidative stress and that induction of Oxr1 may be relevant to neurodegenerative pathways in disease.

## Introduction

Reactive oxygen species (ROS) are the natural by-products of many essential biological processes such as mitochondrial respiration, although they are also potentially damaging to cells. Consequently, eukaryotic organisms have evolved a comprehensive range of proteins to detoxify ROS and repair against any unwanted oxidative damage to DNA, lipids or proteins. These antioxidants include enzymatic scavengers such as superoxide dismutase (SOD) and catalase, glutathione peroxidase (Gpx) and peroxyredoxins, as well as non-enzymatic factors including glutathione, flavonoids and vitamins [1]–[2]. Oxidative stress occurs when the antioxidant response is insufficient to balance the production of ROS; this state can ultimately cause cell death by apoptosis or necrosis via an array of signalling pathways, and many studies both *in vitro* and *in vivo* have demonstrated that the normal function of antioxidant defence systems is vital for cell survival [3]. For example, mouse knockouts representing the most critical mitochondrial antioxidant genes are often lethal at the pre- or early post-natal stage, including glutathione peroxidase 4 (*Gpx4*), thioredoxin 2 (*Thx2*) and SOD2 [4]–[6].

In recent years there has been a particular focus on the role of ROS in neurons, driven by the consistent presence of various oxidative stress markers in neurodegenerative disease, as well as several pathogenic mutations in proteins that feature prominently in antioxidant pathways [3]. Furthermore, it appears that the brain is more vulnerable to ROS damage compared to other organs due to its high metabolic rate combined with a relatively low concentration of antioxidant proteins [7]. Indeed, oxidative stress and mitochondrial dysfunction have been implicated in all major neurodegenerative disorders, including amyotrophic lateral sclerosis (ALS), Parkinson's and Alzheimer's disease (PD and AD) [3], [8]–[9]; yet, despite numerous attempts to recapitulate human disease pathology in mouse models, it is unclear how the timing and disruption of endogenous ROS defence pathways might lead to such heterogeneous neuropathological features [10]. Consequently, with speculation that the up-regulation of antioxidants may be a practical therapeutic target for neurological disease [11], the hunt continues for new proteins that are key players in the oxidative stress response.

In one such search for human factors induced under oxidative stress, Volkert *et al.* identified oxidation resistance 1 (*OXR1*) as a novel gene that was able to suppress DNA damage in *Escherichia coli* oxidative repair-deficient mutants [12]. They went on to report that the human protein, when localised to the mitochondria, was sufficient to prevent oxidative damage in *Saccharomyces cerevisiae* mutants lacking *Oxr1* [13]. Indeed, the gene is found in all eukaryote genomes, although in lower organisms its sequence is restricted predominantly to the highly conserved C-terminal (TLDc) domain [14]. In humans, the TLDc domain-containing gene family is composed of four proteins in addition to OXR1, including nuclear receptor coactivator 7 (NCOA7) and TBC1D24 [14]–[15]. Significantly, a mutation in the TLDc domain of TBC1D24 recently has been found in Familial Infantile Myoclonic Epilepsy (FIME) [16]. The function of this domain has not been established, yet it was originally identified as a catalytic motif [17]. Studies have demonstrated that Oxr1 is induced under oxidative stress [13], [18]; however, virtually nothing is known about this obviously evolutionary significant gene or the TLDc domain itself in mammalian systems.

Here we have used a combination of *in vivo* and *in vitro* approaches to show that the levels of Oxr1 are critical for neuronal survival and that up-regulation occurs in both human disease and mouse models of neurodegeneration. In addition, we demonstrate that the conserved TLDc domain alone is sufficient to confer functionality in the mouse. This study therefore reveals the vital role of Oxr1 in oxidative stress-related neurodegeneration.

## Results

### Bella mutants display cerebellar neurodegeneration

**Figure 1:**
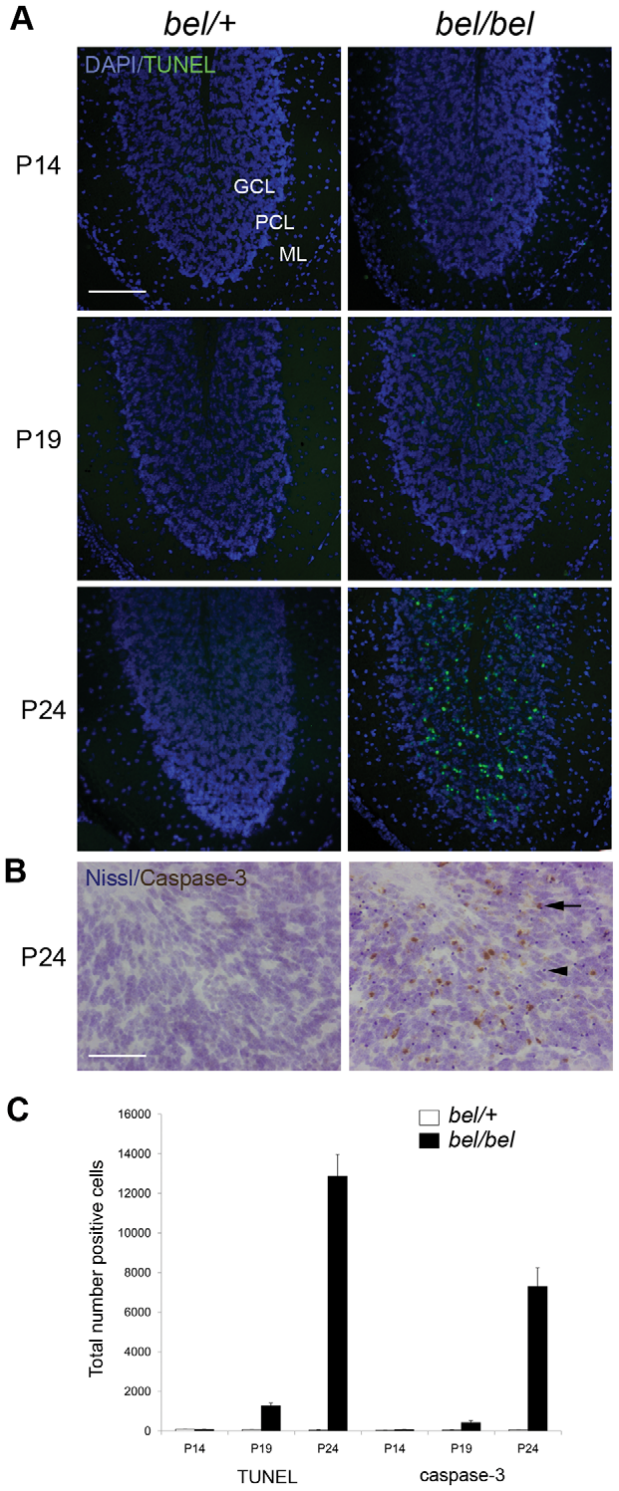
Progressive neurodegeneration in the *bel* mutant. (A) Apoptotic cells are found in the granule cell layer (GCL) but not the Purkinje cell layer (PCL) or molecular layer (ML) of the *bel* (*bel/bel*) cerebellum from P19 as indicated by TUNEL staining. (B) Cleaved caspase-3 immunostaining of *bel* cerebellum at P24 showing positive neurons in the GCL (arrow). Sections are counterstained with cresyl violet (Nissl) indicating condensed nuclei, also indicative of apoptotic cells (arrowhead). (C) Temporal quantification of apoptotic cells in the *bel* and heterozygous (*bel/+*) cerebellum. Mean total number of TUNEL and caspase-3 positive cells from multiple parasagittal sections of the whole cerebellum show a large increase in apoptosis at P24. Scale bars: 150 µM (A), 50 µM (B).

We identified the recessive Bella (*bel*) mouse as part of our screen for mouse models of human movement disorders and ataxia from a large-scale mutagenesis programme. *Bel* mice are indistinguishable from their control littermates at 2 weeks of age (P14); however they rapidly develop a severe ataxic gait (see Video S1), fail to gain weight as quickly as controls, and do not survive beyond P26. Pathological analysis of the *bel* CNS revealed significant and increasing number of apoptotic cells in the granule cell (GC) layer (GCL) of the cerebellum (Figure 1A and 1B). The onset of cell death occurs from P18-19, after which there is a highly significant increase in apoptotic cells in the following days (Figure 1C). No cell death was observed in any other region of the brain or spinal cord in end-stage mutants, however (data not shown). The relative size, structure and foliation pattern of the cerebellum was not affected in late-stage *bel* mutants as determined by quantitative histological methods (Figure S1A, S1D, and S1E) and no significant difference in the GCL width was observed, reflecting the relatively small proportion of apoptotic cells in mutant mice (Figure S1B and S1F). Purkinje cell (PC) death is frequently associated with GC loss [19]–[22] therefore the relative density of PCs was calculated from *bel* mice, although no reduction was observed compared to controls (Figure S1C and S1G). Quantitative histopathology of skeletal muscle was also carried out on end-stage *bel* mice. A significant increase in centrally nucleated fibres was observed in the diaphragm of mutants compared to controls, indicative of muscle degeneration, but not in the tibialis anterior (TA) or soleus muscles of the hindlimb (Figure S1H and S1I). Heterozygous (*bel/+*) mice aged up to 18 months of age display no neuropathological or gait abnormalities (data not shown).

### *Bel* mice contain a 193.5 kb genomic deletion spanning the *Oxr1* gene

An initial genome scan followed by further genetic mapping using polymorphic microsatellite and SNP markers reduced the critical region containing the *bel* mutation to 5.5 Mb on chromosome 15. Unexpectedly, during candidate gene sequencing, exons representing the genes *Oxr1* and Muscle Activator of Rho Signalling (*STARS* or *Abra* [23]) could not be amplified from *bel* DNA. Therefore, genomic walking using chromosomome 15-specific PCR primers followed by inverse PCR was used to identify the boundaries of the apparent spontaneous deletion; the missing region was confirmed as 193.5 kb, ablating the expression of both *Oxr1* and *Abra* (Figure S2A and S2B). To confirm no additional ENU-generated mutation was segregating with the *bel* phenotype, all annotated coding and non-coding transcripts in the critical region were sequenced and no mutations were identified. In addition, qRT-PCR confirmed that the loss of potential regulatory sequences did not influence the expression of all adjacent transcripts within the *bel* critical region (data not shown).

### *Oxr1* is highly expressed in the developing postnatal CNS

**Figure 2:**
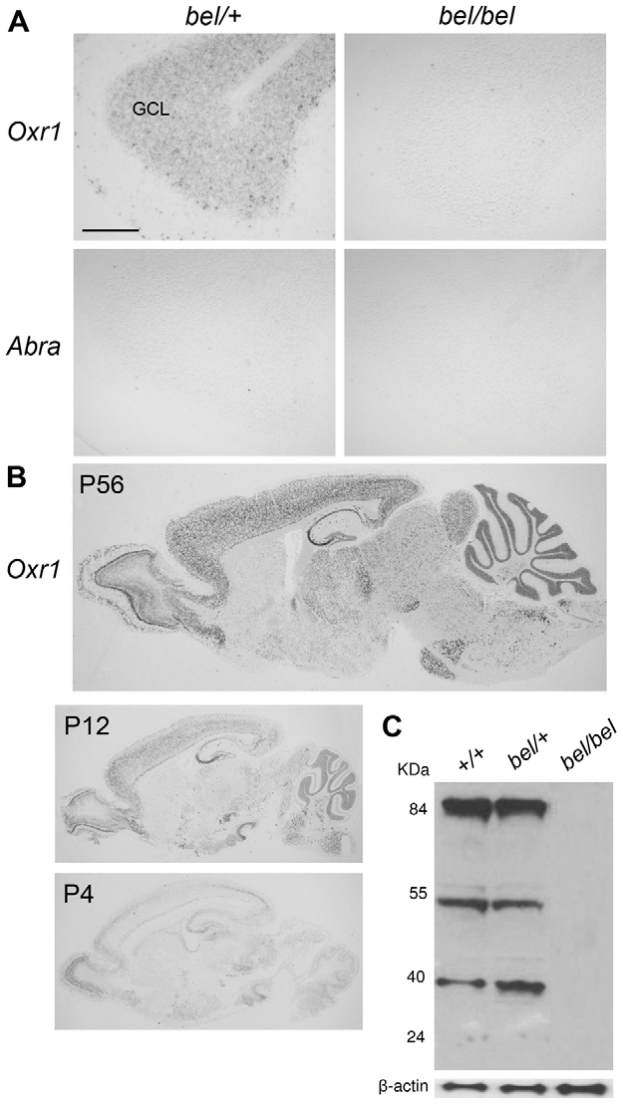
Oxr1 is absent in *bel* mice but highly expressed in the wild-type postnatal brain. (A) *In situ* hybridisation showing the presence of *Oxr1* but not *Abra* in the GCL of the cerebellum in P24 heterozygous (*bel/+*) mice, whereas *bel* mutants lack both genes. (B) *Oxr1* expression in parasagittal sections from wild-type adult and postnatal brain by *in situ* hybridisation. (C) Western blot of Oxr1 from whole brain tissue of mice of each genotype in the *bel* genetic cross; the full-length protein is at approximately 85 kDa and smaller isoforms are observed at approximately 55 and 40 kDa. Note that the specificity of the antibody is also demonstrated by the lack of signal in tissue from *bel* mice. Scale bar: 150 µM (A).

Expression studies were then carried out to determine the distribution of both deleted genes in the central nervous system (CNS). *In situ* hybridisation and RT-PCR showed that while *Oxr1* was expressed in the cerebellar GCL, *Abra* could not be detected in the cerebellum or the rest of the brain (Figure 2A and Figure S3D). Further analysis of the developmental expression patterns showed that *Oxr1* is highly expressed in all major regions of the postnatal brain and spinal cord at the RNA level (Figure 2B and Figure S3A), although *Abra* could only be detected in skeletal muscle tissue by *in situ* hybridisation and RT-PCR (Figure S3B and S3D); these data are consistent with previously published expression data on both genes [13], [23]. In the mouse, several isoforms of Oxr1 have been described, including the shortest isoform that includes only the TLDc domain-containing exons 10 to 16 (or 11 to 16) with a unique first exon (exon 9) (Oxr1-C, also known as C7C [24]; for detail see Figure S7 and Figure S3C). *In situ* hybridisation using isoform-specific probes demonstrated that both the *Oxr1-C* and full-length (*Oxr1-FL*) transcript variants showed an essentially identical expression pattern (Figure S3E), in agreement with the riboprobe common to both isoforms used above (Figure 2B). An antibody raised against the same common C-terminal end of Oxr1 (Figure S7) confirmed high levels of Oxr1 protein in the brain, with no signal in *bel* tissue as expected (Figure 2C); these data also serve to demonstrate the specificity of the antibody. Taken together, these data suggest that loss of *Oxr1* and not *Abra* is responsible for the neuropathology observed in *bel* mutants.

### The *bel* phenotype is rescued by an *Oxr1* transgene

**Figure 3:**
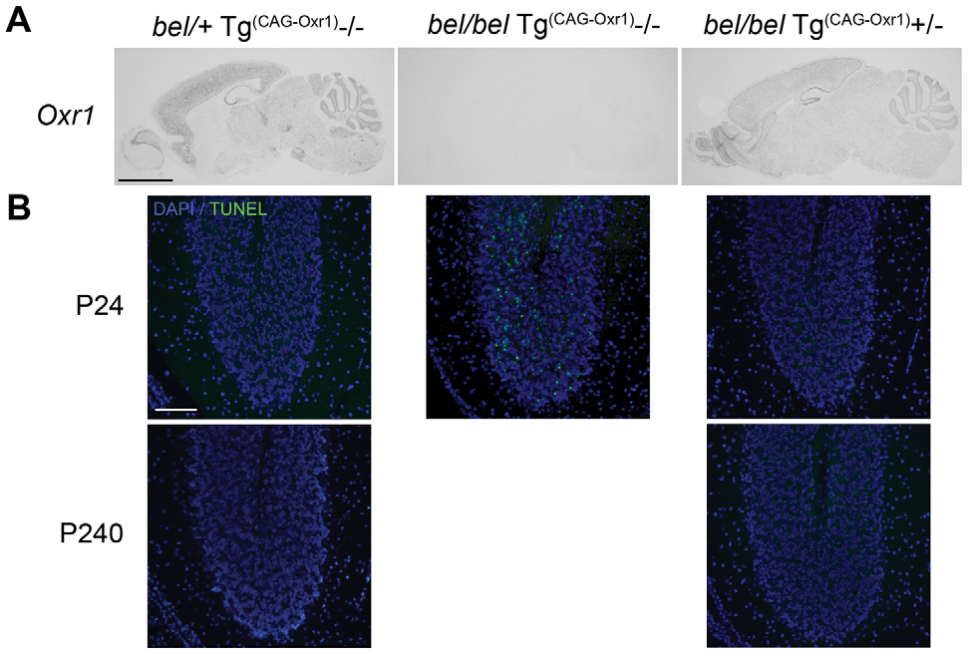
Genetic rescue of the *bel* phenotype with an Oxr1 transgene. (A) *In situ* hybridisation of *Oxr1* from littermates at P24 shows the ubiquitous expression of the Oxr1 transgene (from *bel/bel Tg(Oxr1)/+* sections) in the brain. Two independent lines of *bel/bel Tg(Oxr1)/+* ‘rescue’ mice display no ataxia and no apoptotic cell death in the cerebellum as shown by TUNEL staining (B) compared to control *bel* mice that do not express the transgene (*bel/bel+/+*), demonstrating that replacement of *Oxr1* is sufficient to rescue the *bel* phenotype. Scale bars: 2 mm (A), 150 µM (B).

As conclusive proof that deletion of Oxr1 causes the *bel* phenotype, we performed a genetic rescue experiment with two independent *Oxr1* transgenic lines. Ubiquitous expression of the full-length Oxr1 cDNA (Oxr1-FL) in the brain was confirmed by *in situ* hybridisation in *bel* mutants carrying the transgene (*bel/bel*; Tg^(CAG-Oxr1)^+/−) (Figure 3A). Animals of this genotype displayed no ataxia or growth defects, and no cell death was detected in any region of the brain, including the cerebellar GCL, compared to littermates that did not contain the Oxr1 transgene (*bel/bel*; Tg^(CAG-Oxr1)^−/−) (Figure 3B). This rescue of the *bel* phenotype is maintained in *bel/bel*; Tg^(CAG-Oxr1)^+/− mice aged to 8 months of age (Figure 3B). These data confirm that neurodegeneration in *bel* mice is caused by *Oxr1*.

### Oxr1 levels control the susceptibility of granule cells to oxidative stress

**Figure 4:**
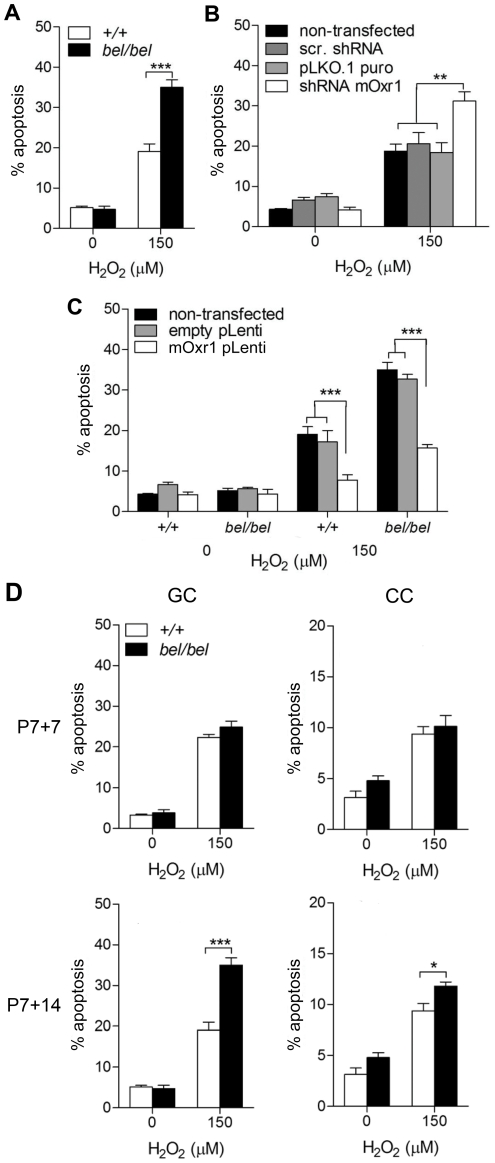
Oxr1 regulates sensitivity of GC neurons to oxidative stress. (A) GCs from P7 *bel* (*bel/bel*) mice cultured for 14 days show a significant increase in apoptotic cell death compared to wild-type (+/+) when subjected to peroxide treatment. (B) Similar results to *bel* GCs are obtained under the same conditions in wild-type GCs transfected with an *Oxr1* shRNA knockdown construct, in contrast with mock transfected (pLKO.1puro) and scrambled shRNA transfected (scr. shRNA) cells. (C) Expression of Oxr1 in *bel* GCs is sufficient to rescue the peroxide sensitivity and protect wild-type GCs from the same treatment. (D) Primary *bel* GCs neurons subjected to H_2_O_2_ treatment demonstrate a relative increase in peroxide sensitivity compared to cortical cells (CC) when cultured to represent the same P14 and P21 timepoint. Note that no significant difference in sensitivity to peroxide treatment is observed in GCs after 7 days of culture between *bel* and wild-type mice, consistent with the lack of cell death observed in *bel* mice at P14. (A–D) Asterisks indicate statistical significance (*P<0.05, **P<0.01 and ***P<0.001; ANOVA).

To demonstrate that loss of Oxr1 rendered neurons from *bel* mice more vulnerable to ROS, primary GCs were assayed for hydrogen peroxide (H_2_O_2_) sensitivity. The assay conditions were first optimised to facilitate measurements of cell death in the presence or absence of Oxr1 (Figure S4A). These data confirmed that *bel* mutant GCs are significantly more susceptible to exogenous peroxide-induced apoptosis than controls (Figure 4A). To further investigate the specificity of this effect, all *Oxr1* isoforms were then knocked-down by an shRNA to <10% of endogenous levels in wild-type GCs, resulting in almost twice the level of cell death compared to neurons transfected with control constructs (Figure 4B and Figure S4B). Conversely, replacement of Oxr1 in *bel* GCs by lentiviral expression rescued the level of apoptotic cell death in H_2_O_2_-treated cells down to wild-type levels (Figure 4C and Figure S4B); thus once again strongly suggesting that loss of Oxr1 alone is responsible for the *bel* phenotype. Significantly, lentiviral over-expression of Oxr1 in wild-type GCs lead to a significant reduction in apoptosis compared to cells expressing endogenous levels of the gene (Figure 4C and Figure S4B), demonstrating that Oxr1 can also be protective to neurons exposed to stress.

In *bel* end-stage mice, apoptotic cell death is specific to the cerebellar GCL, despite high levels of expression in other brain regions including the wild-type cortex (Figure 2B). To therefore investigate whether loss of Oxr1 would also render non-cerebellar neurons susceptible to oxidative damage, we assayed cell death in primary cortical cells (CCs) from *bel* mice in parallel with cerebellar GCs; both cell populations were cultured to correspond to P14 and P21 *in vivo*, respectively (Figure 4D). These data show that no increase in cell death occurs in response to peroxide treatment in *bel* GCs after 7 days in culture; however, after 14 days of culturing a significant increase in apoptosis (approximately 80%) is observed in mutant cells versus wild-type. Although similar results were obtained from CCs, interestingly a much smaller increase in cell death is seen (approximately 20%) in mutants at the second timepoint (Figure 4D). This suggests that in the cerebellum Oxr1 levels play a more defining role in neuronal survival, consistent with the specificity of neurodegeneration in *bel* mice.

Previous studies have described the presence of OXR1 in the mitochondria of HeLa cells [13], but also in the nucleus and nucleolus in other mammalian cell lines using a different antibody [24]. Thus to clarify the localisation of Oxr1 in neuronal cells, immunofluorescence was carried out in wild-type GCs. Using our antibody, Oxr1 was not detectable in GCs unless the cells were treated with H_2_O_2_, which clearly induced protein expression (Figure S4C). In these treated cells, Oxr1 also co-localised with the mitochondrial marker Cox4, consistent with published studies [13]. To determine whether similar induction and localisation was common to other neuronal cell lines, the localisation studies were repeated in N2A cells, generating essentially identical results (Figure S4C). These data demonstrate stress-induction and predominantly mitochondrial localisation of Oxr1 in neurons.

### Loss of Oxr1 induces oxidative DNA damage

**Figure 5:**
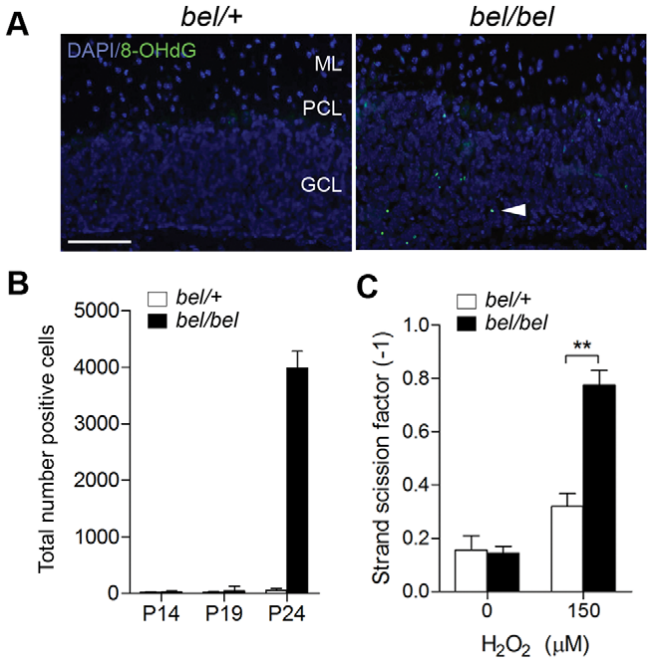
Oxidative DNA damage in *bel* GCs. (A) 8-OHdG immunostaining of cerebellum sections at P24 showing oxidative DNA damage in the GCL of *bel* mice (arrowhead). (B) Temporal quantification of 8-OHdG immunostaining from parasagittal sections of the whole cerebellum showing a large increase in positive cells at P24 in *bel* mice. (C) DNA fragmentation from cultured primary GCs quantified by picogreen. A significant increase in DNA damage is seen in peroxide treated GCs from *bel* mice compared to controls (**P<0.01; ANOVA). Scale bar: 50 µM (A).

In view of the link between Oxr1 and oxidative stress, we then screened for markers of oxidative stress in *bel* mice. 8-OHdG staining was detected exclusively in the mutant GCL at P24, indicative of oxidative DNA damage (Figure 5A). In agreement with the apoptotic markers, virtually no DNA damage was detectable prior to P24 (Figure 5B). To further quantify DNA fragmentation due to loss of *Oxr1*, the DNA strand scission factor from GCs was calculated using a picogreen assay, showing a significant increase in DNA breaks in *bel* GCs subjected to H_2_O_2_ treatment (Figure 5C). We then analysed a large range of both direct and indirect markers of oxidative stress in addition to antioxidant enzymes from the cerebellum of end-stage (P24) *bel* mice by qRT-PCR (Figure S5A). These data identified an approximate 70% reduction in expression of glutathione peroxidase 1 (*Gpx1*) in mutants, although no other genes showed significant differences between the genotypes (Figure S5A and S5B). We went on to test key antioxidants at the protein level, but found no deregulation of the protein expression or enzyme activities of Gpx or catalase in the *bel* cerebellum (Figure S5C, S5D, S5E). Using the same assays, there was no evidence for oxidative stress in brain regions outside of the cerebellum (data not shown). These data combined with the 8-OHdG results suggest that the *bel* cerebellum does show some signs of oxidative stress response due to the loss of Oxr1; although these are clearly limited *in vivo* by the relatively small proportion of neurons affected in end-stage mutant animals.

The cellular and tissue data combined suggest that the effect of Oxr1 deletion is highly specific to GCs in *bel* mice *in vivo*. We therefore investigated whether Oxr1 may also influence sensitivity to other cellular stress factors using serum starvation in cultured GCs. These data show that there was a significant (approximately 7-fold) increase in apoptosis in GC neurons cultured without serum, although no difference in the levels of cell death was observed between *bel* and control GCs (Figure S6). These data suggest that loss of Oxr1 does not influence sensitivity to all cellular stress conditions.

### The conserved TLDc domain of Oxr1 is sufficient to confer protection against oxidative stress

**Figure 6:**
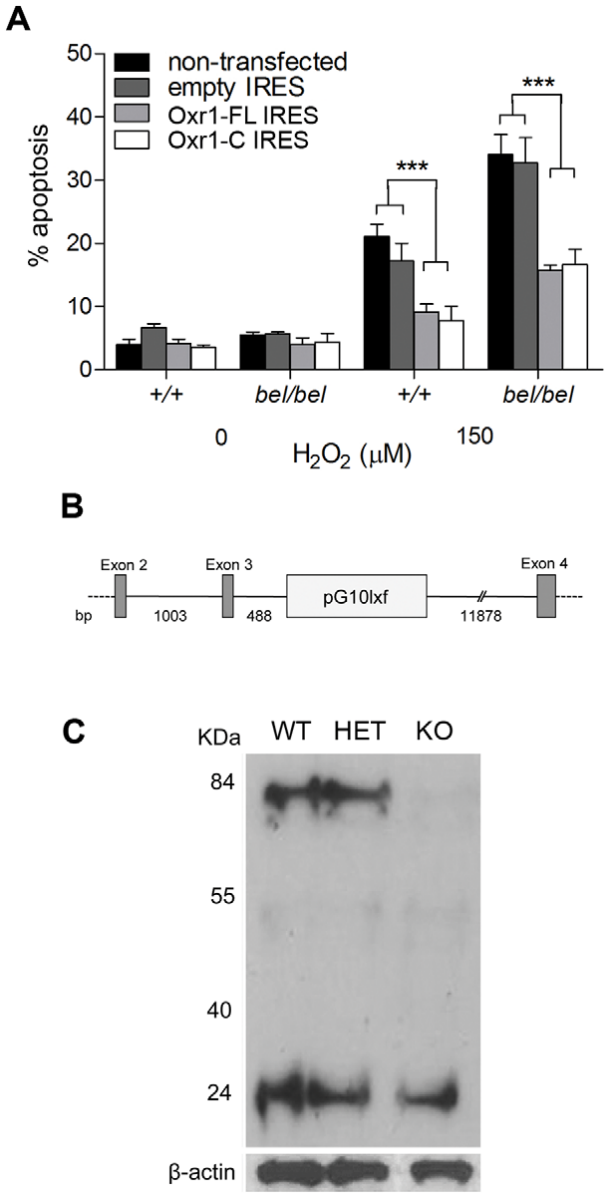
The shortest TLDc domain-containing Oxr1 isoform is able to confer resistance to oxidative stress *in vitro* and *in vivo*. (A) GCs were cultured as above and transfected with Oxr1-FL or –C constructs in an IRES-GFP expression vector. After peroxide treatment, the proportion of cells successfully transfected (GFP positive) that were apoptotic was calculated, showing that both constructs were as effective at preventing cell death in both wild-type (+/+) and *bel* GCs (***P<0.001; ANOVA). (B) Position of the RRR195 gene-trap insertion (pG10lxf vector), 488 bp from the end of Oxr1 exon 3, as determined by PCR. (C) Western blot of cerebellar tissue from littermates in the gene-trap cross showing that in homozygous mice the Oxr1-FL isoform at approximately 85 kDa is barely detectable, whereas Oxr1-C is unchanged.

As discussed above, the C-terminal TLDc domain is highly conserved in all Oxr1 orthologues as well as being highly expressed in the brain (Figure S3D). To therefore investigate whether the short Oxr1-C isoform was functional in neurons, we repeated the peroxide sensitivity assays in *bel* GCs using constructs coding for this isoform as well as Oxr1-FL. In these experiments, a bicistronic vector containing GFP was used to assay the proportion of transfected cells that were apoptotic (Figure 6A). These data show that, despite the removal of over 500 amino-acids from the N-terminus, Oxr1-C is able to confer protection against oxidative stress as efficiently as the full-length protein in both wild-type and *bel* GC culture.

To determine whether the presence of only short Oxr1 isoforms would be detrimental to neuronal survival *in vivo*, a gene-trap mouse (Oxr1^Gt(RRR195)Byg^) was rederived containing the vector insertion between exons 3 and 4 of Oxr1 (Figure 6B and Figure S7); mice carrying two copies of this insertion are therefore expected to only express shorter isoforms of the gene. Mice homozygous for the insertion were successfully generated and the exact position of the trap vector confirmed (Figure 6B). These mice displayed no gait or pathological abnormalities in the CNS up to 12 months of age (data not shown) and western blotting confirmed that the gene-trap insertion had almost completely ablated the expression of Oxr1-FL as expected (Figure 6C). Interestingly, the proportion of the smallest isoform (Oxr1-C at approximately 25 kDa) was much higher in these cerebellar extracts than in whole brain (Figure 6C and Figure 2C); this is consistent with the isoform-specific *in situ* hybridisation data (Figure S3E) and suggests that Oxr1-C may play a more significant functional role in the cerebellum than other regions of the CNS. These data demonstrate that TLDc domain-containing Oxr1 isoforms other than the full-length protein are functional.

### Oxr1 is susceptible to oxidation by peroxide

**Figure 7:**
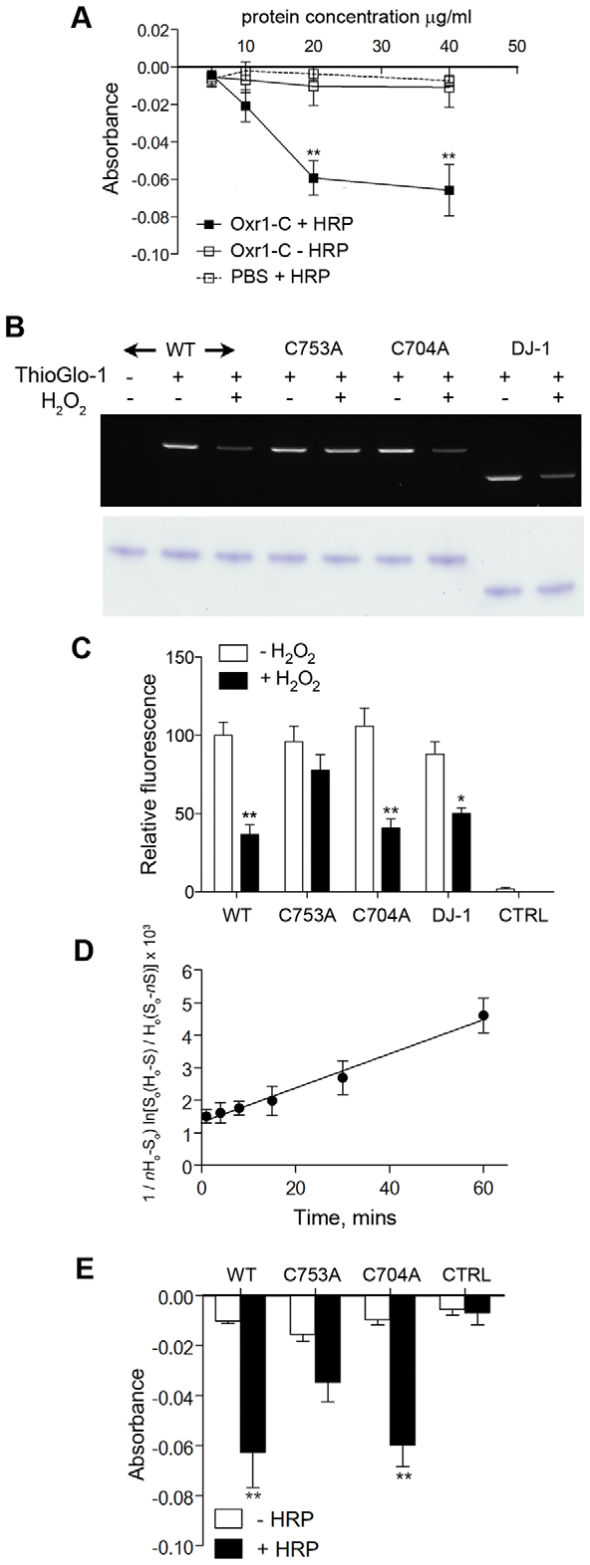
Oxr1 undergoes oxidation by H_2_O_2_. (A) Purified Oxr1-C was incubated with H_2_O_2_ (1.4 µM) and the remaining H_2_O_2_ in the reaction was determined by the absorbance at 580 nm using an Amplex Red assay. The graph shows a reduction in residual H_2_O_2_ with increasing recombinant Oxr1-C concentration; from 20 µg/ml of protein there is a significant decrease in absorbance in the presence of HRP (+ HRP). In the absence of HRP (− HRP) there is no corresponding reduction in absorbance. Control (PBS) reactions contained no protein extract (**P<0.01; ANOVA). (B) Representative in-gel fluorescence of ThioGlo-1 labelled recombinant Oxr1-C proteins and DJ-1. Proteins were subjected or not to H_2_O_2_ treatment as indicated (100 µM, 30 minutes). A significant reduction in fluorescence due to loss of free SH groups by oxidation of cysteine residues was observed in wild-type (WT) Oxr1-C, C704A Oxr1-C and DJ-1, but not the C753A mutant (quantified in (C); *P<0.05; **P<0.01; ANOVA). Control reactions (CTRL) contained no protein extract but included the ThioGlo-1 reagent as a measure of background fluorescence. Lower panel shows staining of the same PAGE gel post-analysis to confirm equal loading. (D) ThioGlo-1 labelling of wild-type Oxr1-C in the presence of H_2_O_2_ was quantified over a time-course to generate a second-order rate constant for the oxidation of Oxr1 (see Materials and Methods for details). (E) The Amplex Red assay was repeated (as panel A) using 20 µg of each recombinant Oxr1-C protein as indicated. No significant reduction in residual H_2_O_2_ was observed when using C753A mutant Oxr1 in the presence of HRP compared to the corresponding – HRP control (**P<0.01; ANOVA). Control reactions (CTRL) contained no protein extract.

To gain some insight for the first time into the mechanism of Oxr1 function, taking into account the results from peroxide stress experiments in GCs, we investigated whether Oxr1 could react directly with H_2_O_2_. Recombinant Oxr1-C protein was purified (Figure S8A) and an Amplex Red assay was used to quantify decreasing H_2_O_2_ concentration in the presence of increasing concentrations of Oxr1-C. These data demonstrate that Oxr1-C is able to significantly decrease the H_2_O_2_ levels in a dose-dependent manner (Figure 7A). As a negative control, the same assay was carried out in the absence of horseradish peroxidase (HRP) that is an essential part of the Amplex Red reaction. These data show no change in Amplex Red signal in the presence of Oxr1-C, suggesting that Oxr1 is not able to compensate for the HRP activity in this assay and is therefore unlikely to possess peroxidase activity.

We next examined whether these data could be due to direct oxidation of the Oxr1 protein. Several amino-acids have the potential to undergo oxidative modification [25], but we began by analysing the oxidation state of cysteine residues considering that a C-terminal cysteine in the TLDc domain is conserved in Oxr1- and Ncoa7-related sequences found in human, mouse, fly and yeast (Cys753 in mouse Oxr1; Figure S8B). To quantify the oxidation of sulfhydryl (SH) side chains, recombinant wild-type Oxr1-C protein was incubated with H_2_O_2_ and then reacted with ThioGlo-1, a thiol-active fluorophore [26]. Samples were then separated by gel electrophoresis followed by densitometric analysis (Figure 7B and 7C). These data show that a significant loss of thiol labelling in Oxr1-C of approximately 2-fold occurs in the presence of H_2_O_2_, indicating that oxidation of free SH groups is taking place. To further examine the significance of cysteine residues in the TLDc domain of Oxr1, the conserved cysteine was mutated (C753A) and the recombinant protein assayed as above (Figure 7B and 7C and Figure S8A). Independently, a second cysteine found in Oxr1 proteins in vertebrates but not the related Ncoa7 was also mutated and analysed (C704A; Figure 7B and 7C, Figure S8A). Peroxide-treated C704A Oxr1-C showed a similar 2-fold reduction in ThioGlo-1 labelling as wild-type Oxr1-C; however, the C753A mutant protein showed a non-significant loss of fluorescence, suggesting that this particular cysteine is more important for the oxidation state of Oxr1 than C704 (Figure 7B and 7C). As a positive control for these studies, DJ-1 (PARK7), a protein that has been well-studied with respect to cysteine oxidation [27]–[28], was analysed in parallel (Figure S8A). Recombinant wild-type mouse DJ-1 showed a similar reduction in ThioGlo-1 labelling upon peroxide treatment to wild-type Oxr1-C (Figure 7B and 7C).

To ascertain the rate of the reaction between wild-type Oxr1 and H_2_O_2_, direct kinetic measurements using HRP competition assays were attempted [29], but the apparent low levels of reactivity between Oxr1-C and peroxide rendered this approach impractical (data not shown). Therefore, ThioGlo-1 labelling experiments were repeated over a time-course, generating a rate constant of Oxr1-C oxidation by H_2_O_2_ of 0.82 M^−1^⋅s^−1^ based on second-order kinetics (Figure 7D). To then relate the significance of the cysteine mutants to consumption of H_2_O_2_ in the Amplex Red assay, both were assayed as above in parallel with wild-type Oxr1-C recombinant protein. In agreement with the thiol labelling assay, the C753A mutant showed a non-significant level of peroxide consumption, whereas a significant drop in fluorescence was observed using the C704A Oxr1-C mutant (Figure 7E). In summary, these data suggest that Oxr1 can react directly with H_2_O_2_, although primarily through the oxidation reactive cysteine residues.

### Oxr1 is over-expressed in ALS and in an ALS model

**Figure 8:**
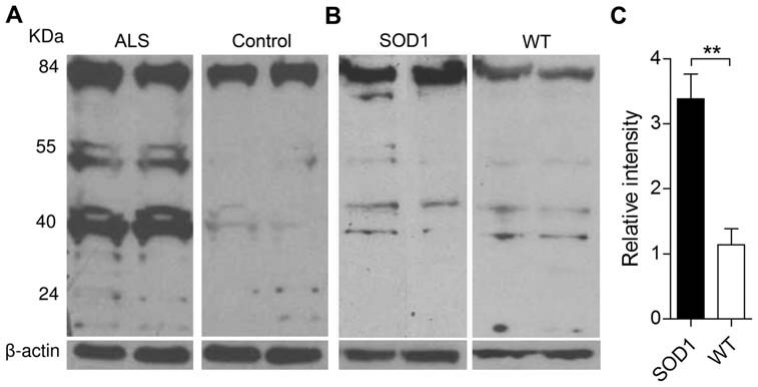
Expression of Oxr1 in ALS and ALS-related neurodegeneration. (A) Western blot showing up-regulation of OXR1 in thoracic spinal cord biopsy samples from ALS patients versus age-matched controls. (B) Representative western blot demonstrating the expression of Oxr1 from the lumbar enlargement of spinal cord tissue from pre-symptomatic SOD1 G93A low-copy transgenic mice and (C) quantification of full-length Oxr1 (85 kDa), *n* = 6 each genotype (**P<0.01; ANOVA).

Considering the numerous links between oxidative stress and neurodegenerative disorders, and the high levels of *Oxr1* in the spinal cord (Figure S3A), we then analysed OXR1 expression in ALS human biopsy samples. Western blots of ALS spinal cord tissue show an obvious up-regulation of the intermediate TLDc-domain-containing OXR1 isoforms compared to age-matched controls (Figure 8A). As these data were obtained from patients at the end-stage of disease, it was also important to ascertain whether up-regulation of Oxr1 occurs before any major neuropathological changes. Therefore, we analysed protein levels in spinal cord tissue from pre-symptomatic low-copy G93A mutant superoxide dismutase 1 (SOD1) expressing transgenic mice, a model of ALS. These data show a significant up-regulation of Oxr1 in SOD1 mutants at 5 months of age compared to littermate controls (Figure 8B and 8C). Importantly, this represents a timepoint prior to the first reported signs of neuropathology or oxidative stress in this particular line [30]–[31], suggesting that Oxr1 may be a novel early marker of specific neurodegenerative pathways.

## Discussion

Combining results from three mutant mouse lines, cellular assays and biopsy samples, we have demonstrated for the first time the importance of Oxr1 in neuronal survival; indeed, our data show that the sensitivity of neurons to exogenous stress can be exquisitely controlled by the level of Oxr1 expression. Oxr1 therefore has much in common with some of the most important antioxidants [3]; proteins such as SOD2 can be lethal when disrupted but neuroprotective when over-expressed *in vivo* and have therefore been nominated as potential therapeutic targets in neurodegenerative disease [6], [32]. Other key mitochondrial proteins have also been linked to ataxia in mouse models. Apoptosis-inducing factor (*Aif*) is vital to oxidative phosphorylation, and an 80% reduction in expression of the gene in the Harlequin (*Hq*) mutant causes ataxia and oxidative stress-related GC loss [33]. The *Hq* phenotype is far less severe than *bel*, however, with the first signs of apoptosis in the cerebellum not appearing until 4 months of age, followed by necrotic Purkinje cell death and degeneration of other brain regions [19], [34].

These models, along with *bel* mutants, emphasise that the brain is clearly vulnerable to oxidative stress. Taken further, the fact that GCs are more susceptible to ROS insults than other neuronal populations, as observed in *bel* and *Hq* mice [33], has been considered recently in detail. Using a combination of expression and biochemical data, Wang *et al.* discovered that cerebellar GCs were more susceptible to exogenous oxidative stress than neurons from the cerebral cortex or hippocampal CA3 region [35]. Lower expression of energy generating genes, combined with a greater depletion of stored ATP, was also observed in GCs versus stress-resistant neurons; this suggested that a shortfall in the energy required to carry out cellular repair might render GCs particularly sensitive to ROS. These data may also explain why we observed that *bel* cortical cells were less sensitive to peroxide treatment than GCs from *bel* mice. Neurodegenerative disease often presents with highly specific pathological lesions despite widespread or ubiquitous expression of the mutated gene(s) involved. Therefore, examples of selective neuronal vulnerability to oxidative stress, such as *bel*, are vital to understand why certain neurons are targeted while others are spared, particularly in the early stages of disease [36].

Several splice variants of Oxr1 have been described previously, although we are the first to show that the shortest of these, Oxr1-C, is able to protect neurons from oxidative stress as efficiently as the full-length (Oxr1-FL) protein. Our western blot data is also the first to demonstrate what appears to be a complex differential distribution of Oxr1 isoforms at the protein level. For example, Oxr1-C (25 kDa) is present in the whole brain at very low levels, although in the cerebellum this splice variant is as highly expressed as Oxr1-FL (85 kDa) whereas other intermediate isoforms are absent. As Oxr1 in the apparently normal gene-trap mouse is almost exclusively represented by the short Oxr1-C protein, we postulate that Oxr1-C plays a more important role in the response of GCs to stress than elsewhere in the CNS. Therefore, loss of this particular isoform as well as the full-length protein, combined with the apparent vulnerability of GCs, leads to cerebellar-specific apoptotic cell death in *bel* mice. Original descriptions of human OXR1 induction by oxidative stress were restricted to intermediate isoforms (at approximately 40 and 58 kDa), based on known splice variants starting upstream of, but including, the TLDc domain [12]–[13]. Although the full-length protein was therefore not addressed in these early experiments, a construct representing the 40 kDa isoform was still able to confer protection against ROS in yeast [13]. Importantly, these original studies are in agreement with our investigation that unequivocally demonstrates that these shorter splice variants, all containing the TLDc domain, are indeed functional.

We chose ALS to model the *in vivo* induction of Oxr1 as oxidative stress has been consistently implicated in the human disease and in ALS mouse models, with multiple markers of oxidative damage observed in ALS post-mortem tissue recapitulated in SOD1 transgenic lines [37]. The striking up-regulation of intermediate OXR1 isoforms we observed in ALS may be a consequence of significant neurodegeneration in the spinal cord; but crucially, full-length Oxr1 protein levels were significantly increased in SOD1 G93A mice before any overt phenotypic abnormalities, suggesting Oxr1 may be an early marker of neurodegeneration [30]–[31], [38]–[39]. The fact that alternate isoforms were differentially regulated between human and mouse may reflect species-specific post-transcriptional regulation of Oxr1 or the dissimilar stages of disease examined.

In summary, it is likely that alternate Oxr1 isoforms have specific purposes; however it is clear from our work that deciphering the function of the highly conserved TLDc domain will be key to understanding the role of Oxr1 and Oxr1-related proteins [14]. In view of this, we assayed recombinant Oxr1-C in a peroxide scavenging assay and discovered that this region of the protein can react directly with H_2_O_2_ *in vitro*. This is the first direct evidence that the TLDc domain, originally predicted to be catalytic [17], may act as an antioxidant protein. Consequently, neurodegeneration in *bel* mice may be due to an increase in ROS that would normally be detoxified by Oxr1. Importantly, however, the calculated rate constant for Oxr1 oxidation by H_2_O_2_ argues against a vital role for the protein as an antioxidant enzyme. Although the value of 0.82 M^−1^⋅s^−1^ for Oxr1 is of the same order of magnitude as reactive cysteine-mediated oxidation of BSA and DJ-1 [28], [40], it is thousands of times lower than key antioxidants such as catalase and peroxiredoxins that have reported oxidative rate constants of over 10^7^ M^−1^⋅s^−1^ [29], [41]–[42]. Therefore, although one attribute of Oxr1 may be to reduce ROS directly, it appears more likely that the oxidation of Oxr1 itself as a consequence of oxidative stress in the cell has a more important functional and/or regulatory role. Such redox-controlled modifications are important for a variety of proteins, including the regulation of conformational changes, often related to the formation or alteration of disulphide bonds [43], [44]. For example, detailed studies of oxidised forms of DJ-1 have focussed on Cys106 as a key residue that mediates the function of the protein, although the mechanistic link between oxidation of this particular amino-acid and the multiple proposed roles for DJ-1 *in vivo* is still unclear [27]. It will therefore be important in the future to ascertain the relationship between our data regarding cysteine oxidation of Oxr1 with three-dimensional structural information of the TLDc domain; for instance, differences in the accessibility of Cys753 and Cys704 to peroxide may go some way to explain the results described here.

A recent study, utilising *Oxr1* knockdown in the mosquito *A.gambiae*, proposed that Oxr1 down-regulates the transcription of the antioxidants catalase and Gpx downstream of the stress-related Jun N-terminal kinase (JNK) [45]. Our transcriptional analysis of oxidative stress-related genes in the *bel* cerebellum also identified a significant reduction in *Gpx1* expression, although this difference was not recapitulated at the protein level or using quantitative enzyme assays. This is most likely due to the small number of GCs affected in end-stage *bel* mice. As the study in *A.gambiae* was limited to transcriptional data, it would be interesting to examine whether the expression changes observed in mosquitoes equate to detectable alterations at the protein level. Although the interaction between Oxr1 and antioxidant enzymes is a plausible functional hypothesis, no mechanism for this particular pathway was investigated. Indeed, our biochemical data suggest that Oxr1 can react directly with ROS, although an indirect influence on other antioxidants, such as *Gpx*, cannot be ruled out.

In the *bel* cerebellum, further evidence for oxidative stress was shown by the large increase in oxidative DNA damage as quantified by 8-OHdG immunostaining. The fact that few markers for the oxidative stress response were altered overall may simply reflect the limited lifespan of the *bel* mutant; only a small proportion of neurons undergo apoptosis before death (approximately 1–2% of all GCs) indicating the relative subtlety of the neuropathology. We can only speculate that if *bel* mice survived for longer whether additional regions of the CNS would be similarly affected. Future work using conditional or inducible disruption of Oxr1 will shed further light on such region-specific mechanisms.

The *bel* mutant is an important new model of oxidative stress-related neurodegeneration, although the short lifespan of mutants limits the study of non-cerebellar neurons *in vivo*. Importantly, however, the fact that Oxr1 is expressed in all major regions of the brain and spinal cord, combined with our data from ALS and SOD1 mutant tissue, suggests that it plays a widespread and vital neuroprotective role. Indeed, it is intriguing that down-regulation of *OXR1* has been recently reported as one of the major differences in a microarray study of the cortex in PD [46]. It is frequently postulated that stimulating endogenous defence pathways would be an effective strategy in combating cell death in disease [9]; our findings therefore provide the first indication that the enhancement of Oxr1 activity *in vivo* may counteract or even prevent the damage carried out by ROS in the progression of neurodegenerative disorders. The apparent functional compensation of OXR1 between yeast and human [13] and the high degree of sequence conservation at particular amino acid residues in the TLDc domain can now be investigated further to help decipher the molecular mechanisms involved. Indeed, it is noteworthy that the alanine mutated in the TLDc domain of the TBC1D24 protein in human FIME is not only conserved in OXR1 (Figure S8B), but has also been shown to inhibit neurite outgrowth *in vitro* [16], suggesting that further study into this family of proteins will also be important for neurological disorders outside of those directly linked with oxidative stress [47].

## Materials and Methods

### Ethics statement

All experiments were performed in accordance with the UK Home Office regulations and approved by the University of Oxford Ethical Review Panel.

### Cloning of the *bel* deletion

The *bel* phenotype was first identified from a screen for recessive ENU mutants at MRC Harwell, UK. To genetically map the trait, 13 *bel* mutants were initially screened for polymorphic SNP markers between the parental C3H/HeH and BALB/c (ENU treated) strains followed by fine mapping using additional microsatellite and SNP markers. Inverse PCR was carried out by digesting *bel* genomic DNA with a range of restriction enzymes, ligating the products, amplifying around the circular DNAs using nested primers and sequencing. A BglII restriction fragment spanning the deletion was consistently identified, which was confirmed using *bel-*specific PCR primers (5′ CGACTAGGCCATCTTCTATTAC and 5′ GCTAATGGCTGCCGAGTTTG). Mice were genotyped using these deletion primers in combination with wild-type control primer (5′ GTGACTGGAGGTGAGCTTTG) or using *D15Mit229*, a polymorphic microsatellite marker in very close proximity to the *bel* deletion.

### *In situ* hybridisation

*In situ* hybridisation was carried out as previously described on 12 µM frozen tissue sections [48]. Regions of *Oxr1* and *Abra* mouse cDNA sequences (see Figure S7) were subcloned into pCR4-TOPO (Invitrogen) prior to DIG-labelled riboprobe synthesis and hybridisation. Slides were exposed for 16 hours in all cases.

### Immunohistochemistry and histology

TUNEL staining for apoptotic cells was carried out on frozen sections using the *in situ* cell death kit (Roche). Antibodies for cleaved caspase-3 (Cell Signalling, 1∶500 dilution, 24 hours at 4°C) and 8-OHdG (QED Biosciences, 1∶250) immunostaining were used on 4% paraformaldehyde perfused, paraffin wax embedded sections; 8-OHdG staining was carried out as previously described [49]. Primary antibody staining was visualised using Vectastain Elite ABC kit (Vectorlabs) or Alexa Fluor 488 or 594 secondary antibodies (Invitrogen) for immunofluorescence.

### Quantitative histopathology

Five 10 µm sections taken at 40 µm intervals from the midline of 3 *bel/+* and 3 *bel/bel* mice were stained with cresyl violet. The total area of each section corresponding to the cerebellum and the remainder of the brain was calculated using Axiovision 4.6 software (Zeiss) and averaged over each genotype. For GCL analysis, the midpoint of lobes III, IV/V and IX was determined as the distance between the apex to the abyss of the fissure. A region representing 0.4 mm, 0.2 mm either side of this midpoint, was used to determine the GCL width by dividing the area of the GCL in this region by 0.4 mm to obtain an average value for each lobe. To examine Purkinje cell numbers, adjacent sections, 5 from each animal, were immunostained using anti-calbindin 28 K (Swant, 1∶15000 dilution, 48 hours at 4°C) as previously described [48]. The total number of Purkinje cells on each section was counted and divided by the total length of the Purkinje cell layer. Adjacent sections to those above were used to count caspase-3 and 8-OHdG immunopositive cells in all cerebellar lobes. Quantification of apoptosis by TUNEL staining was carried out on five 10 µm midline sections at 40 µM intervals from 3 mice of each genotype. For muscle histopathology, tissue samples were dissected and snap frozen in OCT (VWR) on isopentane in liquid nitrogen. Frozen transverse sections were cut at 10 µM for haematoxylin and eosin (H&E) staining using standard methods. Counts of centrally nucleated fibres were averaged from H&E stained sections from 4 mice of each genotype.

### Primary neuronal culture

Culturing of granule and cortical cell cultures was carried out as previously described (Amaxa Nucleofector protocol (Lonza) and Bilimoria *et al.* [50]. *Bel* mutant and control granule or cortical neurons were obtained from postnatal day 7 (P7) or P2 animals, respectively, and cultured for 7 to 19 days prior to treatment. For cell death experiments, cells were treated with 150 µM H_2_O_2_ for 4 hours before being fixed in 4% paraformaldehyde for 15 minutes prior to using the TUNEL assay as above. Cells were assayed for survival by counting 1,500 cells for granule cells or 500 cells for cortical cells. For immunofluorescence, GCs and N2As were treated with 1 mM H_2_O_2_ for 30 minutes prior to recovery in fresh media for 1 hour. Cell counts were analyzed using Prism software; the difference between wild-type and mutant or between the various treatments was compared using ANOVA. *P* values<0.05 were considered significant. All experiments were carried out on 3 or more occasions with cultures obtained from independent mouse litters.

### Expression constructs

For knockdown of *Oxr1* expression, a Mission shRNA construct (Sigma) specific to all TLDc-containing isoforms of the gene (see Figure S7) was used. Primary cells were electroporated with constructs using the Amaxa Nucleofector method (Lonza). The relative level of knockdown was consistently over 90% as shown by qRT-PCR using *Oxr1* exon-spanning primers (Figure S4B). For over-expression of Oxr1, the full-length mouse coding sequence (NM_130885) with a C-terminal HA-tag was cloned into pLenti6/V5-D-TOPO vector (Invitrogen) with a stop codon introduced before the V5 sequence. The constructs were transfected into HEK293T cells with packaging vectors and virus-containing supernatants were collected 3 days later. GCs were infected after 11 days in culture by adding lentivirus-containing medium (1∶50 dilution) and H_2_O_2_ treatment was carried out after 3 days of infection for 4 hours prior to cell survival estimation as above. Lentiviral Oxr1 expression equivalent to endogenous levels were consistently obtained as shown by qRT-PCR using *Oxr1* primers as above (Figure S4B). For over-expression studies comparing Oxr1-FL and Oxr1-C sequences, the coding regions (NM_130885 and NM_001130164, respectively) were cloned into a bicistronic pCAGGS-based vector with additional internal ribosomal entry site (IRES) upstream of GFP. Primary cells were electroporated as above.

### Western blotting

Tissue or cell extracts were prepared using standard RIPA buffer and protein levels were quantified using BSA assays (Pierce Thermo Scientific). After primary antibody (Oxr1 1∶100 (see above); catalase (Abcam); Gpx1 (Epitomics); SOD1 (Abcam)) and peroxidase-conjugated secondary antibody incubation, blots were developed with the ECL kit (Amersham). Frozen thoracic spinal cord samples from non-SOD-related sporadic ALS patients and age-matched controls were obtained from the Thomas Willis Oxford Brain Collection. The lumbar enlargement of the spinal cord from 5-month old male SOD1 G93A mutants (TgN[SOD1*G93A]Gur1) and littermate wild-type controls were dissected and protein extracts prepared immediately as above. Band intensity relative to internal controls was carried out using ImageJ software.

### Quantitative RT–PCR

Expression studies were carried out from total RNA purified using the RNeasy kit (Qiagen). cDNA was generated using Expand Reverse Transcriptase (Roche) and triplicate qRT-PCR reactions carried out using SYBR green (Applied Biosystems). Data were analysed using StepOne software (Applied Biosystems) and normalised to the control β-actin gene in all cases. All data shown are generated from at least 3 independent samples. Primer sequences are shown in Dataset S1.

### Generation of *Oxr1* transgenic line

The full-length mouse Oxr1 coding sequence was cloned into a pCAGGS-derived vector (containing the chicken β-actin promoter with a CMV enhancer and a rabbit β-globin intron), freed of the plasmid backbone by restriction digest and injected into the pronuclei of superovulated CBAB6F1 mice. Founder mice were initially identified using Oxr1 exon-spanning primers for subsequent breeding. Two independent founder females (*Tg^(CAG-Oxr1)^+/*−) were bred to heterozygous *bel/+* males over two generations to generate mice homozygous for the *bel* deletion but also expressing the Oxr1 transgene (*bel/bel*, *Tg^(CAG-Oxr1)^+/−*) to determine genetic rescue. Genetic background effects were controlled by assessing the onset of ataxia and neuropathology of non-transgenic *bel/bel* mutants which proved to be identical to the original *bel* line.

### Generation of the *Oxr1* gene-trap line

Gene-trap ES cell line RRR195 (Oxr1^Gt(RRR195)Byg^) was obtained from Bay Genomics and the correct identity of the insertion was confirmed by RT-PCR from cultured cells prior to rederivation. RRR195 ES cells were injected into preimplantation mouse embryos and chimeras were generated and bred with C57BL/6J mice. Chimera ES cell contribution and germline transmission were assessed by coat colour and confirmed by genotyping. The exact position of the insertion was determined by PCR to generate primers for genotyping; 5′ GTGTTGAGTTCCCCATC and 5′ CCGCAAACTCCTATTTCTGAG for the gene-trap vector or 5′ CAATCTAAATCCACTGCTGAC for the wild-type intron 3/4 control. Mice heterozygous for the insertion were bred together to generate homozygous animals.

### Recombinant Oxr1 protein purification and antibody production

The full-length coding sequence of Oxr1-C (NM_001130164) and a region representing the TLDc domain of mouse Oxr1 (C7C, see Figure S7) were subcloned into the pET-22b(+) expression vector (Novagen) in-frame with a polyhistidine tag (6× His) at the C-terminus. The coding sequence of mouse DJ-1 (NM_020569) was cloned in the same manner. Oxr1 cysteine mutants were generated by QuikChange site-directed mutagenesis (Stratagene) and sequenced prior to use. Constructs were transformed into BL21(DE3) *E.Coli* cells (Invitrogen) and protein expression was induced overnight at 18°C at O.D_600_∼0.8 by addition of isopropyl-β-D-thiogalactopyranoside (IPTG) to a final concentration of 0.1 mM (Oxr1) or 0.25 mM (DJ-1). Bacterial cultures were sonicated and recombinant His-tagged proteins were purified from the soluble fraction using BD Talon metal affinity resin (BD Biosciences Clontech) according to the manufacturer's recommendations. Antiserum was raised in rabbits against the C7C TLDc domain fusion protein (Eurogentec) and affinity purified. For protein oxidation studies, proteins were reduced in 2 mM DTT and subjected to buffer exchange into 50 mM phosphate buffer, pH 7.4 on PD-10 filtration columns (GE Healthcare) prior to use.

### Amplex Red assay

The Amplex Red assay was used to determine the presence and/or depletion of H_2_O_2_ essentially as described by the manufacturer (Molecular Probes). Working solutions of the dye and horseradish peroxidase (HRP) were made fresh for each assay and added to varying amounts of purified recombinant protein. Based on predicted molecular weight of Oxr1-C (28.55 kDa), protein concentrations ranged from 0.175 to 1.4 µM. The reaction was initiated with the addition of H_2_O_2_ at a final concentration of 1.4 µM (e.g. ratio of H_2_O_2_ to Oxr1-C of up to 1∶1). Samples were incubated for 30 minutes at 37°C in the dark and fluorescence readings were obtained at 580 nm. All wells were counted in triplicate correcting for background fluorescence from a blank sample and all experiments were repeated on 3 separate occasions.

### Labelling of free SH groups with ThioGlo-1

Pre-reduced recombinant Oxr1-C protein was incubated with 100 µM H_2_O_2_ (final concentration) at 37°C for up to 30 minutes. Individual aliquots (corresponding to 5 µg of protein) were taken at a range of timepoints, excess H_2_O_2_ was removed with purified catalase (Sigma), and the amount of reactive thiols determined by incubation with 30 µM ThioGlo-1 for 90 minutes at 60°C. Samples were mixed with Laemmli loading dye (without bromophenol blue to prevent background fluorescence) and subjected to SDS-PAGE. Gels were visualised with an ultraviolet light source and subjected to densitometry using a Fluor-S MultiImager (BioRad). To confirm equal loading of protein, gels were post-stained using SimplyBlue (Invitrogen). The rate constant for this reaction was estimated by plotting (1/*n*H_o_−S_o_) ln[S_o_(H_o_−S)/H_o_(S_o_−*n*S)] versus time as previously described [40], where H_o_ represents the initial concentration of H_2_O_2_, S_o_ is the initial concentration of free SH groups, S is the SH content reacted and *n* is the moles of free SH oxidised per mole of H_2_O_2_; *n* was taken to be a value of 2 given two potential reactive cysteines in the Oxr1-C recombinant protein fragment used.

### Catalase and Gpx assays

Catalase activity from cerebellar tissue samples was carried out using Amplex Red Catalase Assay Kit (Molecular Probes) and Gpx activity was measured using the Glutathione Peroxidase Assay Kit (Calbiochem), both according to the manufacturer's instructions based on standard curves of enzyme activity.

### DNA fragmentation assay

Genomic DNA from cells was extracted using the Genomic DNA Extraction Kit for tissues (Qiagen). Double-stranded DNA was quantified using PicoGreen (Thermo Scientific) as per manufacturer's instructions and compared to a standard curve generated from λ/HindIII DNA to determine a ratio of dsDNA to ssDNA (strand scission factor) in the sample.

## Supporting Information

Dataset S1 Primers used for qRT-PCR of oxidative stress markers.
(DOC)

Figure S1 Quantitative histological analysis of the *bel* cerebellum and muscle. (A) Cresyl violet staining of a vermal parasagittal section indicates that no disruption in the foliation of the *bel* cerebellum occurs. A small but non-significant reduction in cerebellar size is observed in *bel* mice compared to controls, based on area calculations from multiple sections (D). The size of the *bel* cerebellum in not proportionally smaller than controls, however (E). (B) Cresyl violet staining of lobe IV/V indicating the position of the cerebellar granule cell (GCL), molecular (ML) and Purkinje cell (PCL) layers. A small but non-significant reduction in average GCL width is observed in *bel* mice compared to controls in lobes III, IV/V and IX; data for lobe IV/V are shown (F). (C) Adjacent sections were immunostained with anti-calbindin and used to calculate the average Purkinje cell density in *bel* mice. There was no difference in density between the genotypes showing no cell death in the PCL in *bel* mice (G). (H) Haematoxylin and eosin staining of representative transverse sections of *bel* and wild-type (+/+) diaphragm muscle indicating centrally nucleated fibres in mutants (arrowheads). (I) Quantification of centrally nucleated fibres in the soleus, TA and diaphragm from all genotypes (**P<0.01, ANOVA). Scale bars: 0.5 mm (A) and 0.1 mm in (B) and (C).
(TIF)

Figure S2 Identification of the *bel* deletion. (A) Annotation of mouse chromosome 15 showing the critical mapped genetic region for the *bel* phenotype. The exact position of the deletion as determined by inverse PCR as indicated, and confirmed by PCR using flanking primers (B).
(TIF)

Figure S3 Additional expression analysis of *Oxr1* and *Abra*. (A) *In situ* hybridisation showing expression of *Oxr1* but not *Abra* in wild-type spinal cord at P24. The same *Abra* riboprobe detects expression of the gene in skeletal muscle (B). (C) Gene structure of full-length Oxr1 (Oxr1-FL) and short Oxr1 (Oxr1-C) isoforms, not to scale; exon 9 is unique to Oxr1-C. (D) RT-PCR of the entire protein-coding sequence of *Oxr1-FL*, *Oxr1-C* and *Abra* from P24 brain (lanes labelled B) and cerebellum (lanes labelled C) tissue. Skeletal muscle (M) is also shown as a positive control for *Abra* expression. Note that the two bands amplified using *Oxr1-C* primers correspond to transcripts either containing (*Oxr1-C*) or lacking (*Oxr1-C′*) the alternatively spliced exon 10. Negative control reactions from template containing no RT enzyme are indicated (−). (E) *In situ* hybridisation of adult (P56) mouse brain using riboprobes specific to *Oxr1-FL* and Oxr1-C. For details of the relative probe positions, see Figure S7.
(TIF)

Figure S4 Primary neuronal culture control data. (A) Gradient of H_2_O_2_ treatment tested in primary GCs to generate a robust stress response for apoptotic cell counts. (B) Quantitative RT-PCR of relative total *Oxr1* expression level after knockdown and lentiviral over-expression from 3 independent experiments. (C) Immunostaining of GCs and N2A cells using the Oxr1 antibody demonstrates induction and co-localisation of Oxr1 with the cox4 mitochondrial marker 1 hour after peroxide treatment.
(TIF)

Figure S5 Oxidative stress markers in *bel* cerebellum. (A) Quantitative RT-PCR from end-stage *bel* and wild-type littermate cerebellum tissue for a range of oxidative stress makers: catalase (*cat*), cullin1 (*cul1*), cytochrome c (*cytc*), glutamate-cysteine ligase catalytic and modifier subunits (*gclc*/*gclm*), glial fibrillary acidic protein (*gfap*), glutathione peroxidise 1 (*gpx1*), heme oxygenase 1 (*ho1*), kelch-like ECH-associated protein 1 (*keap1*), NAD(P)H dehydrogenase, quinone 1 (*nqo1*), nuclear factor (erythroid-derived 2)-like 2 (*nrf2*), Peroxisome proliferator-activated receptor gamma coactivator 1-alpha (*pgc1α*), superoxide dismutase 1 and 2 (*Sod1* and *Sod2*). Data are also shown as relative expression ratio between genotypes for key antioxidant enzymes (B), including a significant reduction in *gpx1* expression in mutants (*** *P*<0.01, ANOVA). (C) No difference in the protein levels of catalase, Gpx1 or SOD1 is observed in cerebellar tissue between end-stage *bel* and littermate control mice as shown by western blot. Enzyme assays also show no difference in the activity of Gpx (D) or catalase (E) from the same tissue.
(TIF)

Figure S6 Serum starvation of GCs. Primary GCs from wild-type and *bel* mice were cultured for 14 days were subjected to serum starvation for 4 hours. No difference in apoptosis was observed between genotypes.
(TIF)

Figure S7 Diagram of cDNAs and probes used. cDNA structures of mouse Oxr1-FL and Oxr1-C (not to scale) with relative positions of probes/antibodies used in this study. The structure of the gene-trap cDNA is also shown.
(TIF)

Figure S8 Protein purification of Oxr1 and C-terminal sequence alignment. (A) Coomassie staining of SDS-PAGE gels showing the induction (left) and subsequent purification (right, 5 µg loading) of wild-type and mutant histidine-tagged Oxr1-C and DJ-1 proteins. The position of the mutated cysteine amino acids in the TLDc domain refers to full-length mouse Oxr1 protein sequence (accession number NP_570955). (B) ClustalW alignment of the C-terminal region of the TLDc domain in Oxr1-related proteins from *Homo sapiens* (hs), *Mus musculus* (mm), *Xenopus laevis* (xl), *Danio rerio* (dr), *Drosophila melanogaster* (dm) and *Saccharomyces cerevisiae* (sc). Position of the cysteine residue C753 in mouse Oxr1 is marked. The alanine residue in TBC1D24 recently reported as mutated in human FIME (A509V) is highlighted in yellow.
(TIF)

Video S1 Film demonstrating the phenotype of the *bel* mutant. A *bel* male mouse at P24 is shown, later joined by a heterozygous (*bel/+*) littermate.
(WMV)
