# Supplementary material for: Oxr1 Is Essential for Protection against Oxidative Stress-Induced Neurodegeneration
Source: PLoS Genet. 2011 Oct 20;7(10):e1002338. doi: 10.1371/journal.pgen.1002338 (PMC3197693; doi:10.1371/journal.pgen.1002338)
Supplement: Dataset S1 — Primers used for qRT-PCR of oxidative stress markers. (DOC) [file pgen.1002338.s001.doc]

Dataset S1 for Oliver et al. PGENETICS-D-11-01031R1

Catalase: 5'-ACCCTCTTATACCAGTTGGC and 5'-CATGCACATGGGGCCATCA; cullin 1: 5’-TGGAAAGCCTGAAGCTCAAT and 5’-TATTTGGCACGAACACCTGA; cytochrome c: 5’-GCAAGCATAAGACTGGACCAAA and 5’-TTGTTGGCATCTGTGTAAGAGAATC; DJ-1: 5’-TTGCACTAGCCATTGTGGAG and 5’-ACATACAGACCCGGGATGAG; glutamate-cysteine ligase catalytic and modifier subunits: 5’-ACATCTACCACGCAGTCAAGGACC and 5’-CTCAAGAACATCGCCTCCATTCAG; glial fibrillary acidic protein: 5’-CGAGTCCCTAGAGCGGCAAATG and 5’-CGGATCTGGAGGTTGGAGAAAGTC; glutathione peroxidise 1: 5’-GTCTCTCTGAGGCACGATCGG and 5’-TTCCGCAGGAAGGTAAACAGC; heme oxygenase 1: 5’-CAAGCCGAGAATGCTGAGTTCATG and 5’-GCAAGGGATGATTTCCTGCCA; kelch-like ECH-associated protein 1 5’-AAGGACCTTGTGGAAGACCA and 5’-CCCTGTCCACTGGAATTGAT; NAD(P)H dehydrogenase, quinone 1: 5’-GCGAGAAGAGCCCTGATTGTACTG and 5’-TCTCAAACCAGCCTTTCAGAATGG; nuclear factor (erythroid-derived 2)-like 2: 5’-TTCTTTCAGCAGCATCCTCTCCAC and 5’-ACAGCCTTCAATAGTCCCGTCCAG; Peroxisome proliferator-activated receptor gamma coactivator 1-alpha: 5’-CCGTGACCACTGACAACGAG and 5’-GCTGCATGGTTCTGAGTGCTAAG; superoxide dismutase 1: 5’-CAAGCGGTGAACCAGTTGTG and 5’-TGAGGTCCTGCACTGGTAC; and superoxide dismutase 2: 5’-GCCTGCACTGAAGTTCAATG and 5’-ATCTGTAAGCGACCTTGCTC.
