# Supplementary figures and images for: Oxr1 Is Essential for Protection against Oxidative Stress-Induced Neurodegeneration
Source: PLoS Genet. 2011 Oct 20;7(10):e1002338. doi: 10.1371/journal.pgen.1002338 (PMC3197693; doi:10.1371/journal.pgen.1002338)

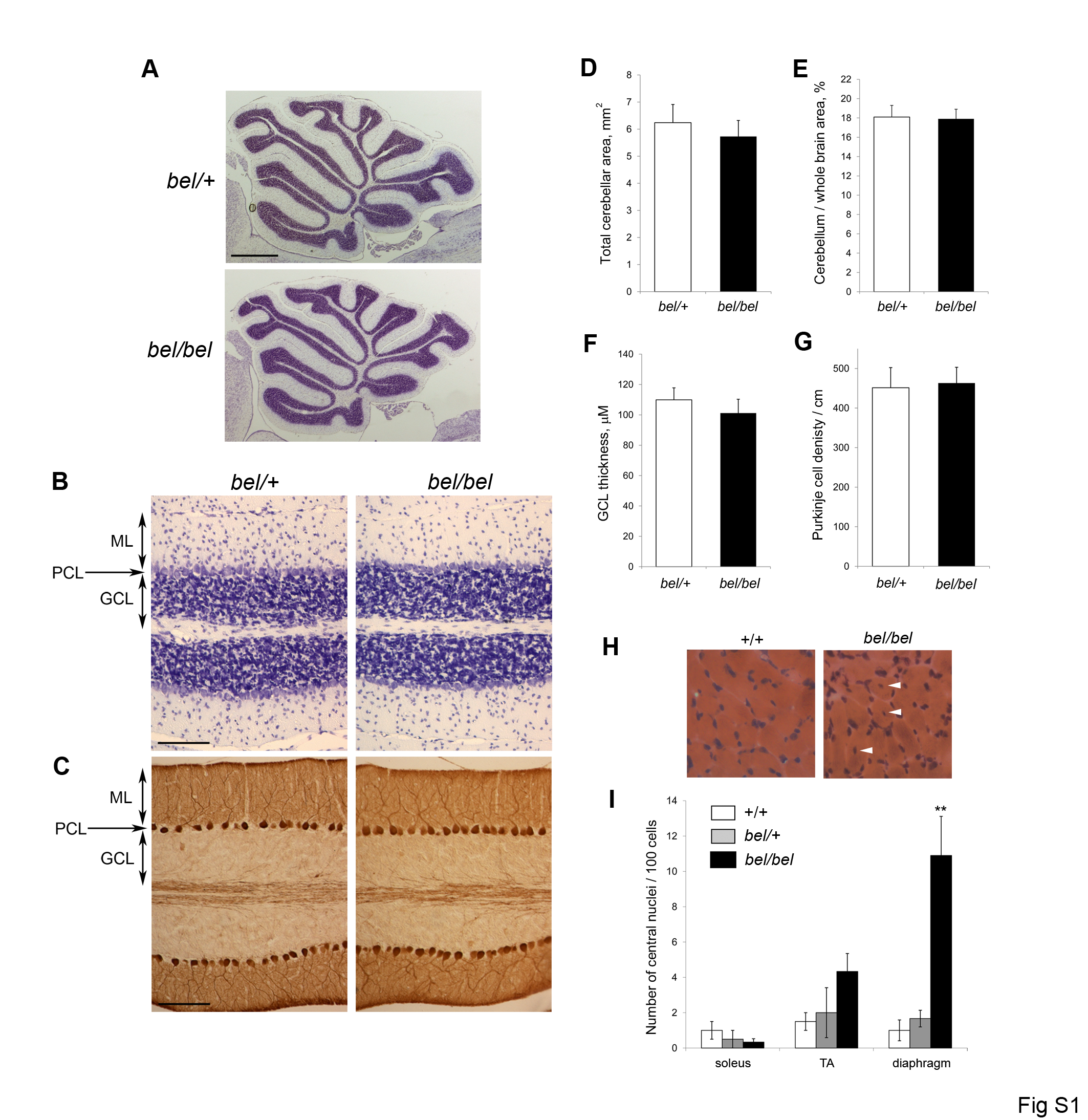

Supplement: Figure S1 — Quantitative histological analysis of the bel cerebellum and muscle. (A) Cresyl violet staining of a vermal parasagittal section indicates that no disruption in the foliation of the bel cerebellum occurs. A small but non-significant reduction in cerebellar size is observed in bel mice compared to controls, based on area calculations from multiple sections (D). The size of the bel cerebellum in not proportionally smaller than controls, however (E). (B) Cresyl violet staining of lobe IV/V indicating the position of the cerebellar granule cell (GCL), molecular (ML) and Purkinje cell (PCL) layers. A small but non-significant reduction in average GCL width is observed in bel mice compared to controls in lobes III, IV/V and IX; data for lobe IV/V are shown (F). (C) Adjacent sections were immunostained with anti-calbindin and used to calculate the average Purkinje cell density in bel mice. There was no difference in density between the genotypes showing no cell death in the PCL in bel mice (G). (H) Haematoxylin and eosin staining of representative transverse sections of bel and wild-type (+/+) diaphragm muscle indicating centrally nucleated fibres in mutants (arrowheads). (I) Quantification of centrally nucleated fibres in the soleus, TA and diaphragm from all genotypes (**P<0.01, ANOVA). Scale bars: 0.5 mm (A) and 0.1 mm in (B) and (C). (TIF) [file pgen.1002338.s002.tif]

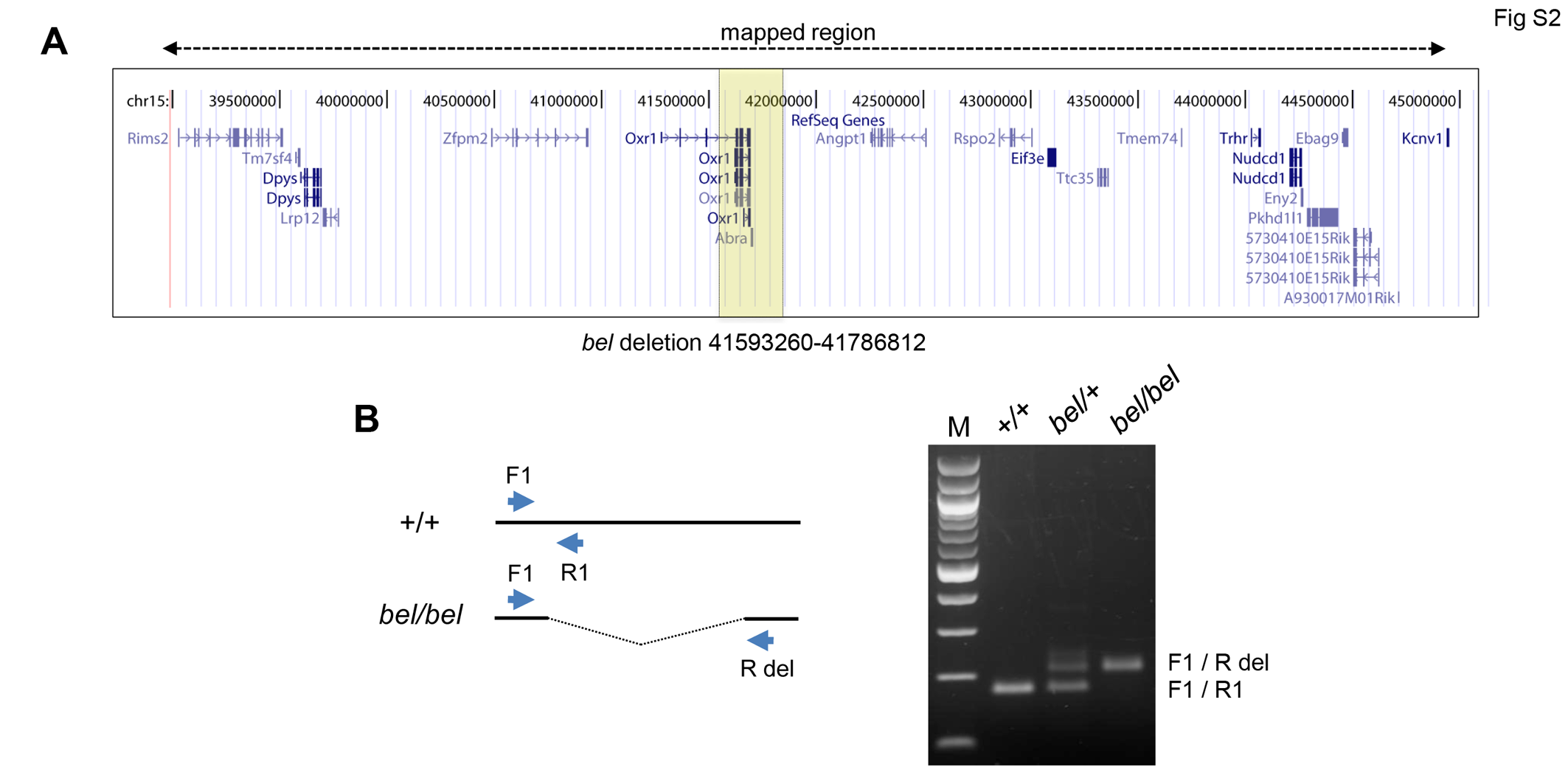

Supplement: Figure S2 — Identification of the bel deletion. (A) Annotation of mouse chromosome 15 showing the critical mapped genetic region for the bel phenotype. The exact position of the deletion as determined by inverse PCR as indicated, and confirmed by PCR using flanking primers (B). (TIF) [file pgen.1002338.s003.tif]

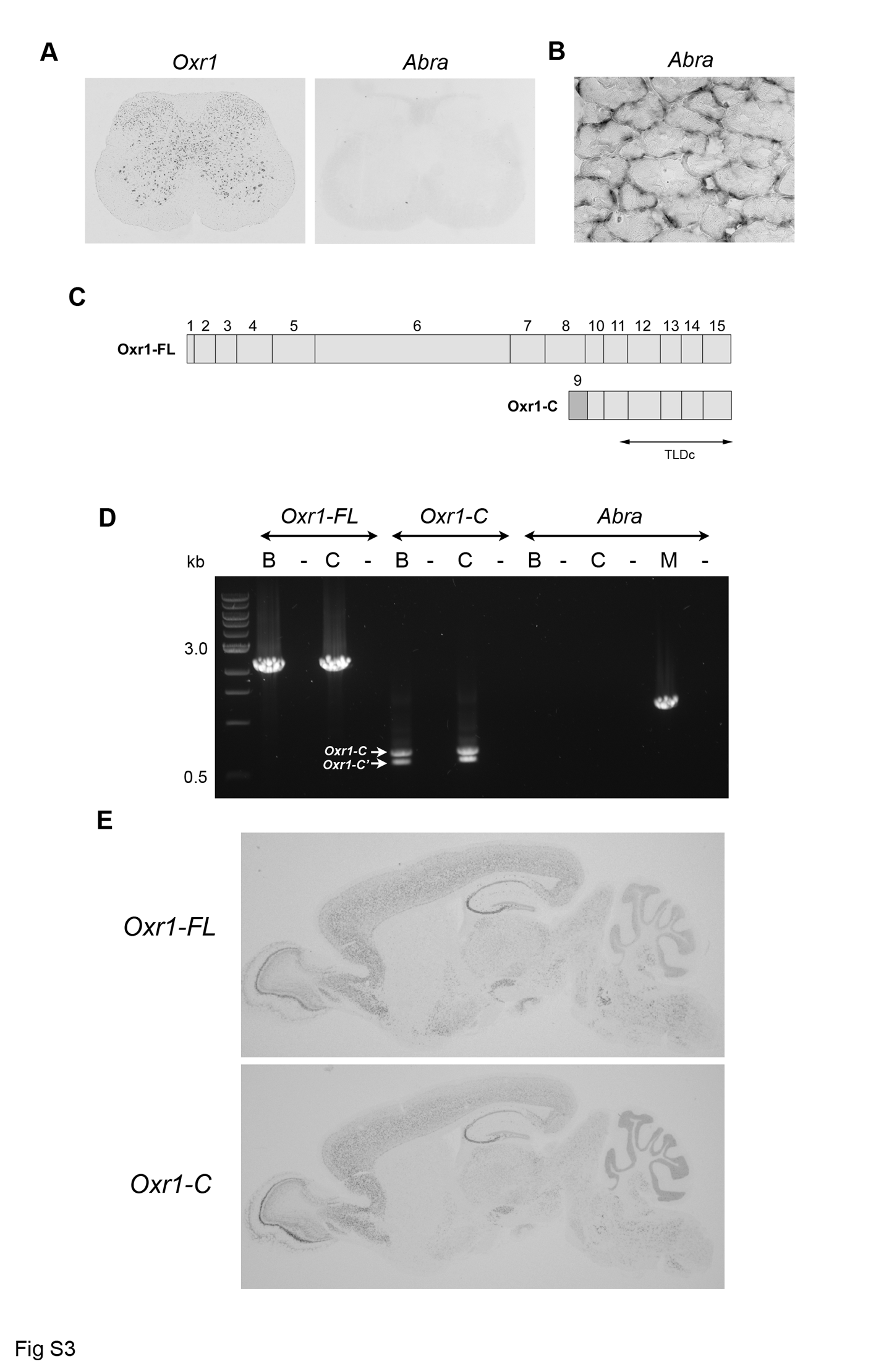

Supplement: Figure S3 — Additional expression analysis of Oxr1 and Abra. (A) In situ hybridisation showing expression of Oxr1 but not Abra in wild-type spinal cord at P24. The same Abra riboprobe detects expression of the gene in skeletal muscle (B). (C) Gene structure of full-length Oxr1 (Oxr1-FL) and short Oxr1 (Oxr1-C) isoforms, not to scale; exon 9 is unique to Oxr1-C. (D) RT-PCR of the entire protein-coding sequence of Oxr1-FL, Oxr1-C and Abra from P24 brain (lanes labelled B) and cerebellum (lanes labelled C) tissue. Skeletal muscle (M) is also shown as a positive control for Abra expression. Note that the two bands amplified using Oxr1-C primers correspond to transcripts either containing (Oxr1-C) or lacking (Oxr1-C′) the alternatively spliced exon 10. Negative control reactions from template containing no RT enzyme are indicated (−). (E) In situ hybridisation of adult (P56) mouse brain using riboprobes specific to Oxr1-FL and Oxr1-C. For details of the relative probe positions, see Figure S7. (TIF) [file pgen.1002338.s004.tif]

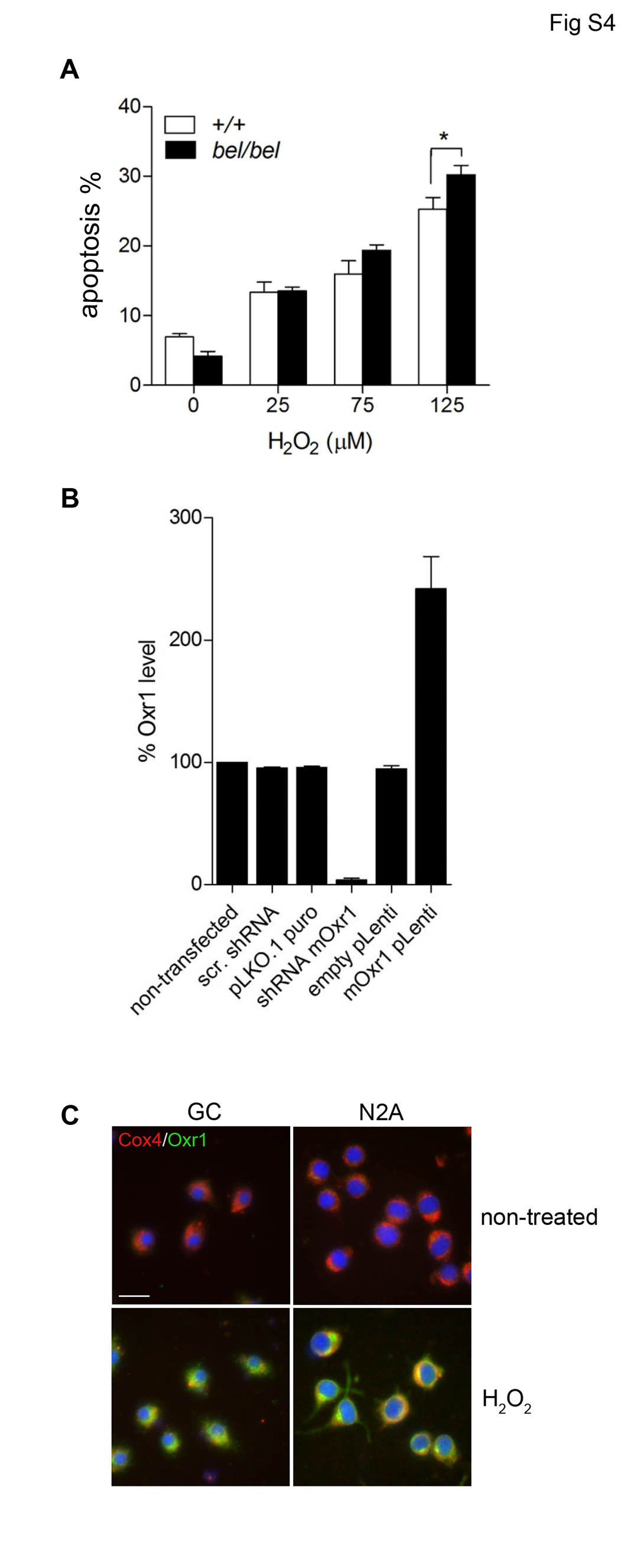

Supplement: Figure S4 — Primary neuronal culture control data. (A) Gradient of H2O2 treatment tested in primary GCs to generate a robust stress response for apoptotic cell counts. (B) Quantitative RT-PCR of relative total Oxr1 expression level after knockdown and lentiviral over-expression from 3 independent experiments. (C) Immunostaining of GCs and N2A cells using the Oxr1 antibody demonstrates induction and co-localisation of Oxr1 with the cox4 mitochondrial marker 1 hour after peroxide treatment. (TIF) [file pgen.1002338.s005.tif]

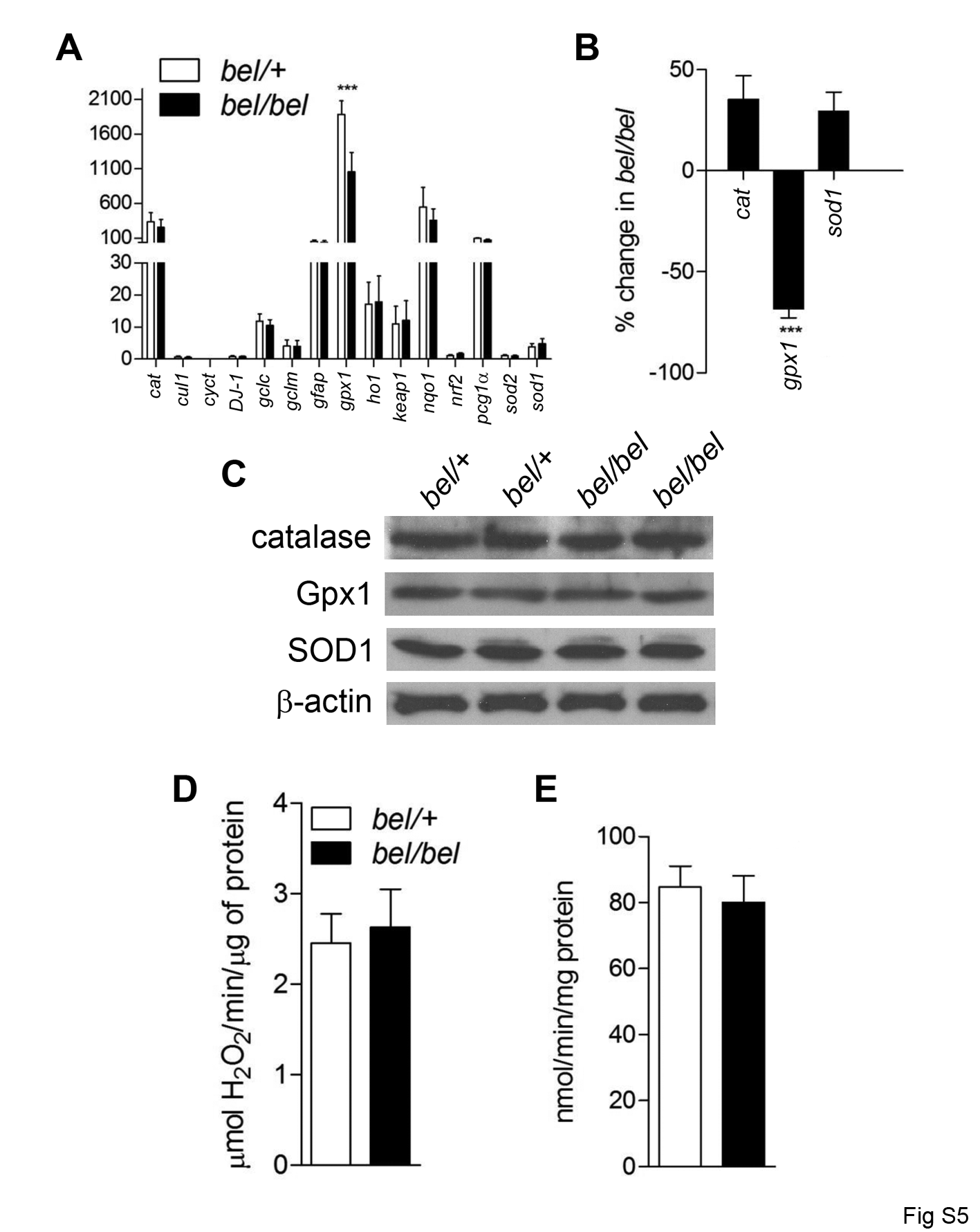

Supplement: Figure S5 — Oxidative stress markers in bel cerebellum. (A) Quantitative RT-PCR from end-stage bel and wild-type littermate cerebellum tissue for a range of oxidative stress makers: catalase (cat), cullin1 (cul1), cytochrome c (cytc), glutamate-cysteine ligase catalytic and modifier subunits (gclc/gclm), glial fibrillary acidic protein (gfap), glutathione peroxidise 1 (gpx1), heme oxygenase 1 (ho1), kelch-like ECH-associated protein 1 (keap1), NAD(P)H dehydrogenase, quinone 1 (nqo1), nuclear factor (erythroid-derived 2)-like 2 (nrf2), Peroxisome proliferator-activated receptor gamma coactivator 1-alpha (pgc1α), superoxide dismutase 1 and 2 (Sod1 and Sod2). Data are also shown as relative expression ratio between genotypes for key antioxidant enzymes (B), including a significant reduction in gpx1 expression in mutants (*** P<0.01, ANOVA). (C) No difference in the protein levels of catalase, Gpx1 or SOD1 is observed in cerebellar tissue between end-stage bel and littermate control mice as shown by western blot. Enzyme assays also show no difference in the activity of Gpx (D) or catalase (E) from the same tissue. (TIF) [file pgen.1002338.s006.tif]

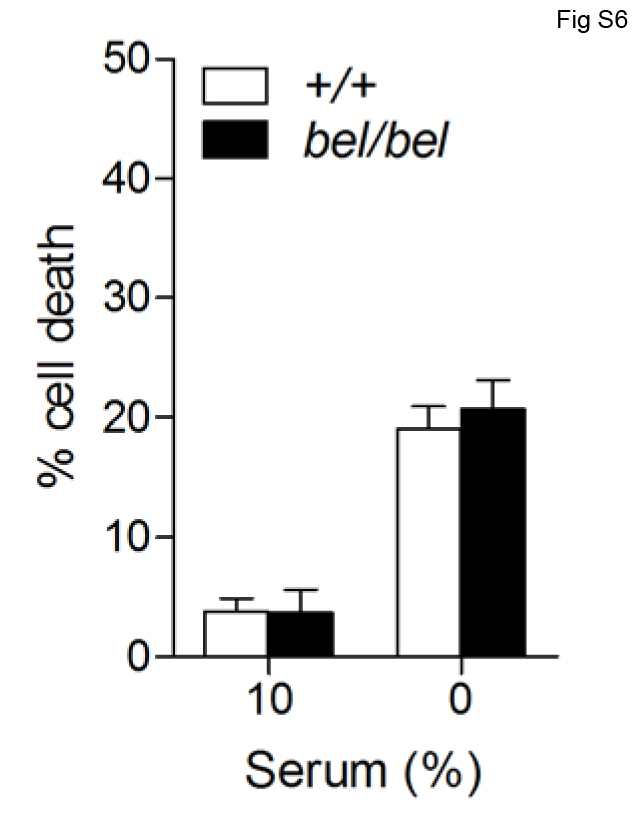

Supplement: Figure S6 — Serum starvation of GCs. Primary GCs from wild-type and bel mice were cultured for 14 days were subjected to serum starvation for 4 hours. No difference in apoptosis was observed between genotypes. (TIF) [file pgen.1002338.s007.tif]

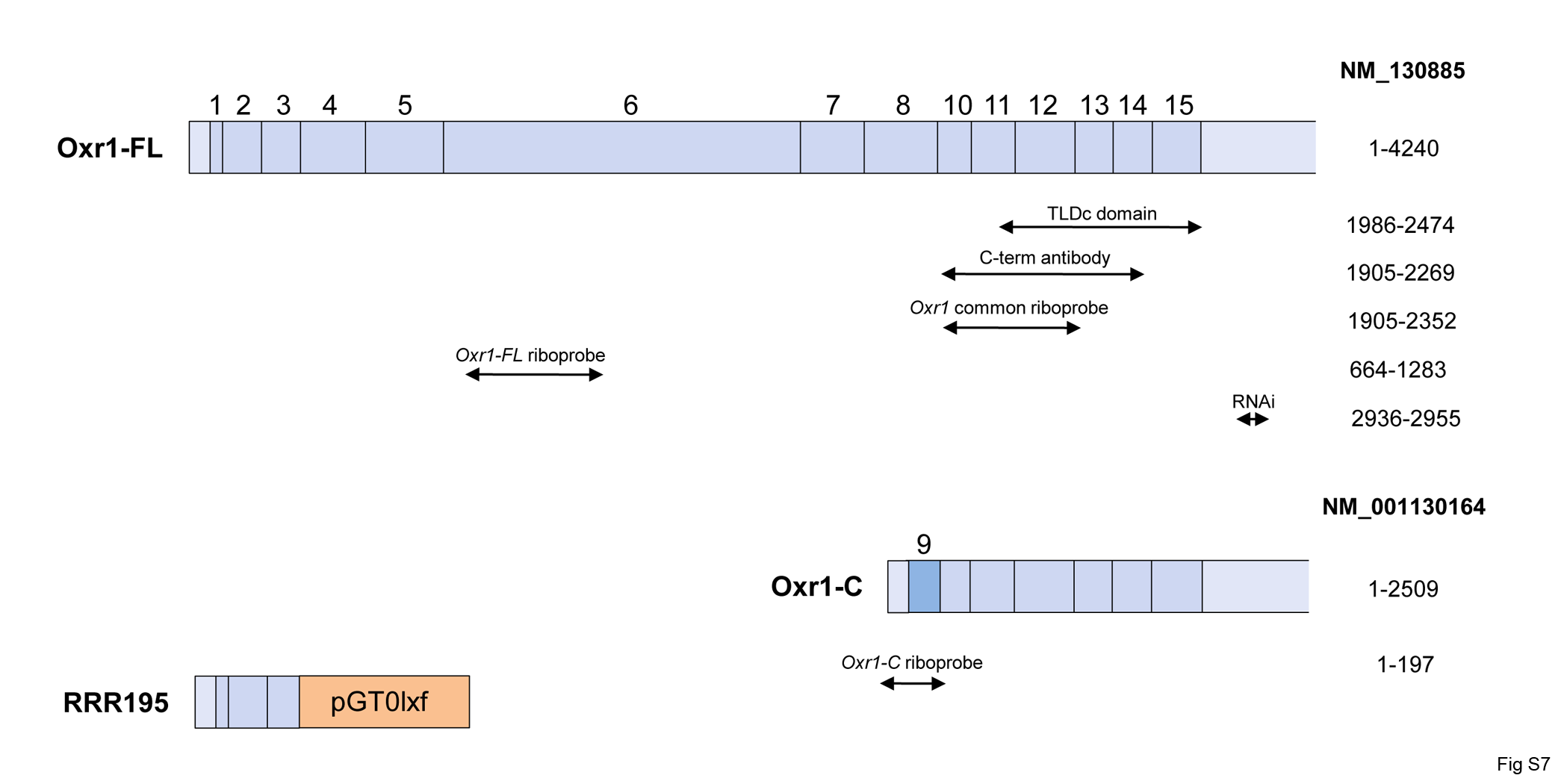

Supplement: Figure S7 — Diagram of cDNAs and probes used. cDNA structures of mouse Oxr1-FL and Oxr1-C (not to scale) with relative positions of probes/antibodies used in this study. The structure of the gene-trap cDNA is also shown. (TIF) [file pgen.1002338.s008.tif]

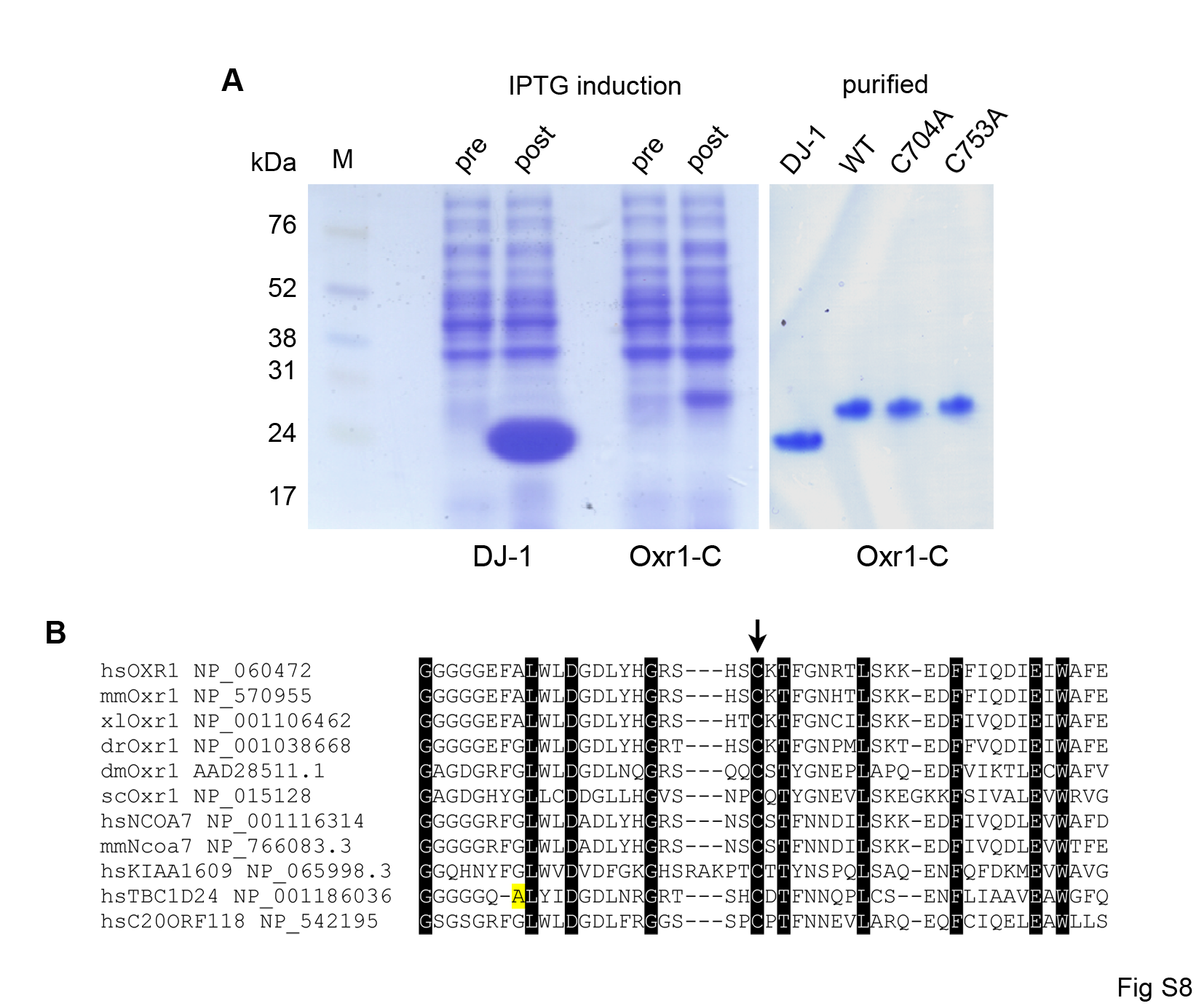

Supplement: Figure S8 — Protein purification of Oxr1 and C-terminal sequence alignment. (A) Coomassie staining of SDS-PAGE gels showing the induction (left) and subsequent purification (right, 5 µg loading) of wild-type and mutant histidine-tagged Oxr1-C and DJ-1 proteins. The position of the mutated cysteine amino acids in the TLDc domain refers to full-length mouse Oxr1 protein sequence (accession number NP_570955). (B) ClustalW alignment of the C-terminal region of the TLDc domain in Oxr1-related proteins from Homo sapiens (hs), Mus musculus (mm), Xenopus laevis (xl), Danio rerio (dr), Drosophila melanogaster (dm) and Saccharomyces cerevisiae (sc). Position of the cysteine residue C753 in mouse Oxr1 is marked. The alanine residue in TBC1D24 recently reported as mutated in human FIME (A509V) is highlighted in yellow. (TIF) [file pgen.1002338.s009.tif]
